# NsrM (All0345) and NsrX (Alr1976), two FurC (PerR)-targeted transcriptional regulators, modulate nitrogen metabolism and heterocyst differentiation genes in the cyanobacterium *Anabaena* sp. strain PCC 7120

**DOI:** 10.1128/spectrum.02311-25

**Published:** 2025-10-13

**Authors:** Jorge Guío, Marta Acero, Anindita Bandyopadhyay, Deng Liu, Himadri B. Pakrasi, Isabelle Michaud-Soret, M. Teresa Bes, Emma Sevilla, María F. Fillat

**Affiliations:** 1 Departamento de Bioquímica y Biología Molecular y Celular e Instituto de Biocomputación y Física de Sistemas Complejos (Bifi), Universidad de Zaragoza 16765, Zaragoza, Spain; 2 Department of Biology, Washington University 123752, St. Louis, Missouri, USA; 3 IRIG/CEA-Grenoble, Grenoble, France; Connecticut Agricultural Experiment Station, New Haven, Connecticut, USA

**Keywords:** cyanobacteria, transcriptional regulators, regulatory network, nitrogen metabolism, Ferric uptake regulator C (PerR), NtcA

## Abstract

The control of nitrogen metabolism in the model cyanobacterium *Anabaena* sp. strain PCC7120 is a complex process orchestrated by master regulators such as NtcA, HetR, and FurC (PerR). These proteins establish complex networks with secondary regulators, finely tuning cellular metabolism in response to diverse, often undefined environmental signals. The XRE-like Alr1976 and the MerR-like All0345 proteins are two predicted transcriptional regulators regulated by FurC. While All0345 is widespread, both proteins have homologs conserved across several bacterial phyla, with *alr1976* often followed by a gene encoding a Zn-metalloprotease. Previous transcriptomic analyses showed that *furC* overexpression affected *alr1976* expression slightly more under nitrogen-deficient conditions, while changes in *all0345* expression were exclusively observed under N deficiency, pointing them as critical candidates for understanding the finer details of nitrogen control in *Anabaena*. This work shows that both Alr1976 (NsrX) and All0345 (NsrM) are potentially modulated by NtcA and work as nitrogen secondary regulators. Electrophoretic mobility shift assays and transcriptomic analyses of ∆*nsrX* and ∆*nsrM* deletion strains indicate that both regulators act as repressors of key genes involved in nitrogen metabolism and heterocyst development. Notably, the ∆*nsrM* strain showed earlier heterocyst formation at 24 h of nitrogen step-down. NsrX and NsrM display distinct requirements for optimal DNA-binding activity to nitrogen metabolism genes (presence of Mn and reducing environment, respectively), suggesting they respond to different environmental stimuli. This differential signal integration likely enables master regulators FurC and NtcA to exert more precise control over shared targets, thereby refining the intricate network of nitrogen metabolic regulation in *Anabaena*.

**IMPORTANCE:**

Filamentous, nitrogen-fixing cyanobacteria are valuable organisms for biotechnology applications and as models for the study of multicellularity in prokaryotes. Understanding the regulation of nitrogen fixation and heterocyst development is essential for optimizing their use in synthetic biology and as biofertilizers. This study identifies two novel nitrogen secondary regulators, Alr1976 (NsrX) and All0345 (NsrM), as part of the intricate regulatory circuit governing nitrogen metabolism in the model cyanobacterium *Anabaena* sp. strain PCC7120. Genes encoding NsrX and NsrM are regulated by both FurC (PerR) and NtcA, therefore taking part in the NtcA-PerR network that modulates nitrogen metabolism and heterocyst differentiation genes.

## INTRODUCTION

Cyanobacteria are prevalent organisms in diverse and often harsh environments thanks to their remarkable adaptability, including metabolic flexibility to vary nitrogen acquisition strategies. The preferred source of nitrogen in cyanobacteria is ammonia, as it can be directly incorporated into carbon skeletons via the GS-GOGAT cycle (1). Although cyanobacteria contain specific ammonia permeases (Amt), the main source of ammonia is the metabolization of other forms of nitrogen, mainly the assimilation of nitrate and nitrite, and the metabolism of simple organic molecules such as urea or amino acids (2). Besides, if these sources are not available, diazotrophic cyanobacteria are capable of fixing atmospheric nitrogen (1).

Due to the oxygen sensitivity of nitrogenase, nitrogen fixation in filamentous cyanobacteria, such as *Anabaena* sp. (also known as *Nostoc* sp.) strain PCC 7120, is spatially segregated into specialized cells called heterocysts. Heterocysts create a microoxic environment by ceasing oxygen-evolving photosystem II activity, increasing respiration, and developing protective envelopes. This differentiation is tightly regulated by metabolic signals, including 2-oxoglutarate (2-OG), and a cascade of transcriptional regulators, the most important of which are NtcA and HetR (3). However, in the last years, several transcriptional regulators aside from NtcA and HetR have been identified as important players in heterocyst differentiation, including the ferric uptake regulator (FUR) paralogues FurA/Fur, FurB/Zur, and FurC/PerR. FurA is a global regulator in *Anabaena*, controlling iron homeostasis, the oxidative stress response, the modulation of nitrogen metabolism, and heterocyst differentiation (4, 5).

Interestingly, FurA and NtcA are interactive regulators, integrating the regulation of iron homeostasis, redox status, and nitrogen metabolism (6). FurA was also found to be able to work as a carbon/nitrogen balance sensor via 2-OG (7) and to bind to the promoter of *hetR* and genes involved in heterocyst formation, such as *hetC* or *patA* (4, 5). Among other processes, FurB (Zur) controls zinc homeostasis and metal trafficking and is involved in the modulation of biofilm formation (8, 9). Notably, a *furB* deletion strain exhibits altered expression of genes, such as *cnifK*, *hetP*, and *patA*, affecting heterocyst frequency (9). FurC corresponds to the peroxide response regulator (PerR) in *Anabaena* sp. strain PCC 7120 (10). Transcriptomic studies unveiled that FurC is a global regulator that modulates cell division and PSII recycling (11). Furthermore, FurC plays a key role in heterocyst differentiation, as its overexpression impairs this process (12). In fact, FurC was found to directly control genes involved in all the steps of heterocyst differentiation, including patterning and early differentiation (*hetZ* and *asr1734*), HEP and HGL formation (*hepC*, *alr4973-75*), and N_2_ fixation (*nifH2*, *nifHDK*, *xisHI*, and *rbrA*) (12). Interestingly, other genes involved in heterocyst differentiation, such as *patA* or *henR*, are differentially expressed in the *furC* overexpression strain, but FurC was not able to bind to their promoter region (12).

Recent work highlights FUR proteins as cornerstones of transcriptional regulatory networks in *Anabaena* sp. strain PCC 7120, controlling transcriptional regulators, two-component systems, serine/threonine kinases, and sigma factors (13). This implies that FUR proteins modulate gene expression not only directly, but also indirectly via secondary regulatory genes. Among them, *all0345* and *alr1976*, encoding potential transcriptional regulators, were found to be directly regulated by FurC (13). According to transcriptional changes in a *furC*-overexpressing variant, FurC acts as an activator of *all0345* and as a repressor of *alr1976* (12). Interestingly, changes in the expression of *all0345* were only observed under N deficiency (fold change +2.5), whereas in the case of *alr1976*, transcriptional changes were slightly higher under N deficiency conditions than under standard conditions (i.e., −3.9-fold and −3.7-fold, respectively) (12). Given the significant role of FurC in the regulation of heterocyst formation, the functions of its downstream targets, Alr1976 and All0345, warrant further investigation. Therefore, we explored their biochemical properties and potential involvement in the control of nitrogen metabolism. Our results suggest a role of Alr1976 and All0345 as secondary regulators in the modulation of nitrogen assimilation and heterocyst differentiation in *Anabaena* sp. strain PCC 7120. Considering their biochemical features and our experimental findings, we propose naming Alr1976 as NsrX (Nitrogen secondary regulator, XRE-like) and All0345 as NsrM (Nitrogen secondary regulator, MerR-like).

## RESULTS

### Bioinformatic analyses of Alr1976 (NsrX) and All0345 (NsrM)

**Fig 1:**
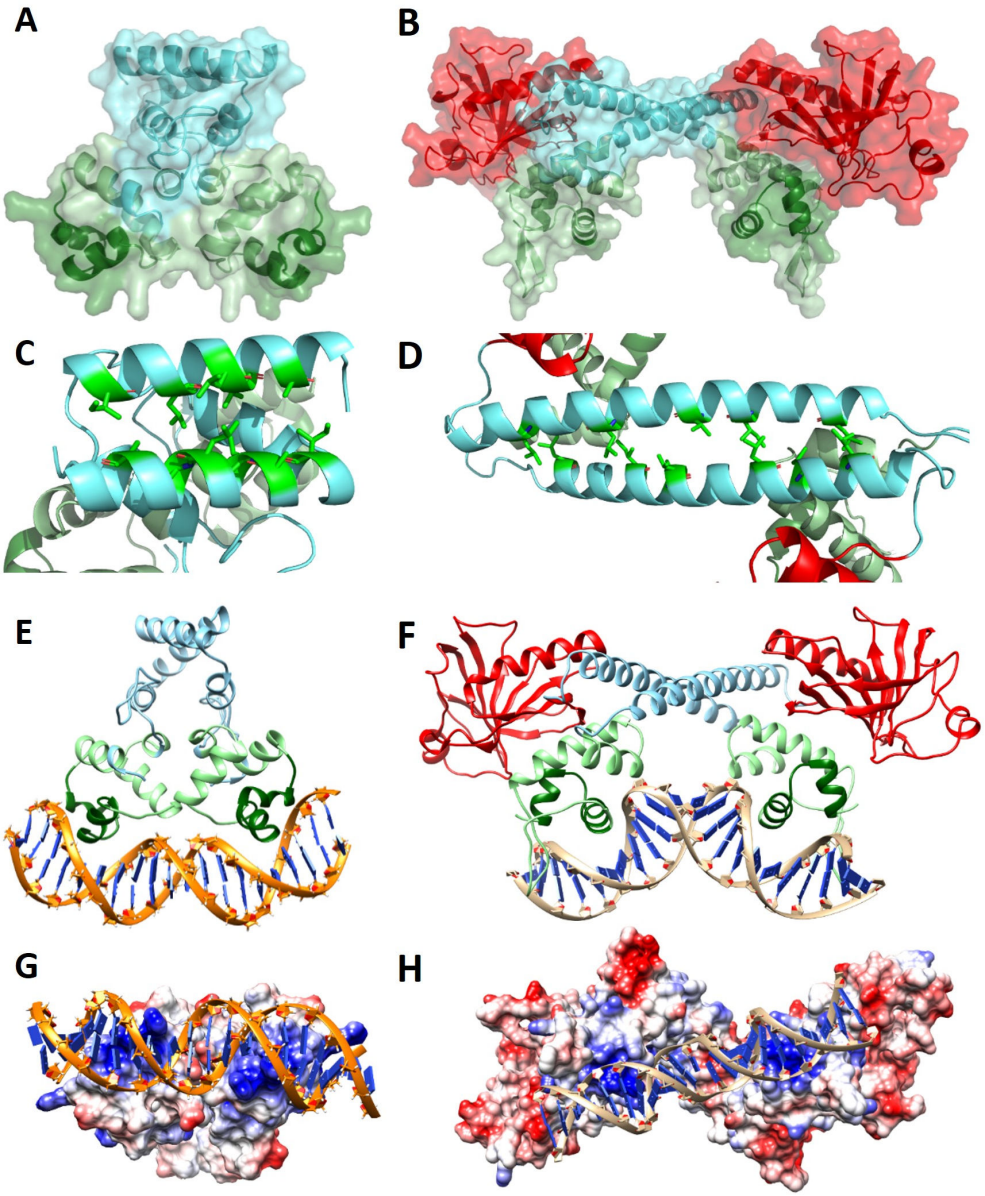
Homology modeling of Alr1976 (NsrX) and All0345 (NsrM), showing the predicted dimer structures and models for protein-DNA interaction. Prediction of dimers of the proteins NsrX (**A**) and NsrM (**B**) using the Swiss-Model server. In both cases, the surface is shown with 50% transparency to visualize the secondary structure motifs. The DNA-binding domain is indicated in green, with the HTH motif highlighted in dark green. In the case of the NsrM protein, the AraC-E-type effector-binding domain is also indicated in red. (**C, D**) Visualization of the hydrophobic residues present in the α-helices involved in the dimerization of NsrX (**C**) and NsrM (**D**). (**E, F**) Models of protein-DNA complexes for NsrX (**E**) and NsrM (**F**). The DNA-binding domain is indicated in green, with the HTH motif highlighted in dark green. In the case of NsrM, the AraC-E-type effector-binding domain is also indicated in red. (**G, H**) Electrostatic surfaces for NsrX-DNA and NsrM-DNA complexes. Positive charge is represented in blue, while negative charge is shown in red.

Initially, a bioinformatic study was carried out to better understand the biochemical properties of Alr1976 and All0345 (hereafter referred to as NsrX and NsrM). Searches in Prosite, Interpro, and SMART unveiled that NsrX belongs to the XRE (xenobiotic-response element) family of transcriptional regulators. Members of this family typically work as repressors binding to their target promoters as dimers (14). These regulators share an N-terminal DNA-binding domain composed of five α-helices, with the second and third helices constituting the HTH motif, while the C-terminal region is highly variable and is involved in signal sensing or dimerization (15). In the case of All0345, bioinformatic studies revealed the presence of a highly conserved HTH DNA-binding domain of the MerR type in the N-terminal region, followed by a central alpha helix and a C-terminal effector-binding domain of type AraC-E (16) (Fig. 1A). Members of the MerR family can modulate transcription in response to metals, oxidative stress, or metabolites (17). They dimerize through the central alpha helix and generally act as activators in response to a specific signal (18) (Fig. 1B).

Secondary and tertiary structure predictions of Alr1976 and All0345 were performed using PSIPred and AlphaFold, respectively (Fig. S1). These predictions confirmed that both proteins contain the characteristic α-helical regions involved in DNA binding and dimerization. Alr1976 exhibited a well-defined HTH DNA-binding domain in the N-terminal region and two α-helixes in the C-terminal region. All0345 displayed a HTH DNA-binding domain in the N-terminal region, whereas the AraC-E effector-binding domain was predicted to have an α/β folding. Both domains are connected by a long α-helix with a flexible hinge, which could facilitate conformational changes upon ligand binding.

To further investigate their structural properties, homology models of Alr1976 and All0345 were built using Swiss-Model (Fig. 1). For Alr1976, we used the *Pseudomonas aeruginosa* regulator BswR (PDB: 4O8B) as template, which showed 18.81% sequence identity and 83% coverage (15). The model for All0345 was based on the structure of the *Escherichia coli* regulator EcmrR (PDB: 6XLK), showing 29.77% sequence identity and 90% coverage (19). In both cases, dimer formation was predicted, consistent with known structures of the XRE and MerR regulators. The predicted dimeric interfaces involved α-helixes with internal faces rich in hydrophobic residues that could mediate protein-protein interactions (Fig. 1C and D). Besides, since protein-DNA complexes have been resolved or modeled for the selected templates, the dimer models of Alr1976 and All0345 were aligned onto these complexes to generate protein-DNA interaction models. In both cases, the predicted dimers were compatible with DNA binding (Fig. 1E through H).

### Conservation of NsrX and NsrM in bacteria

A screen for homologs was conducted using STRING v.11.5. Homologs for Alr1976/NsrX were found in 6 of the 21 bacterial groups (Fig. S2). NsrX was present in Actinobacteria, Cyanobacteria, and Chloroflexi. Among cyanobacteria, NsrX was present in a few strains, both nitrogen-fixing and non-fixers, mostly from freshwater habitats (Fig. S3). According to MicrobesOnline (http://www.microbesonline.org/), *alr1976*/*nsrX* is predicted to be part of a dicistronic operon together with *alr1977*, which, according to KEGG and Pfam, encodes a Zn-metalloprotease homolog with an N-terminal IrrE domain that takes its name from the central regulator of DNA damage repair in *Deinococcaceae* (20). Interestingly, STRING analysis unveiled that the arrangement of an XRE-family regulator followed by this kind of metalloprotease is conserved in several bacterial groups (Fig. S4), including cyanobacteria. A similar study of All0345/NsrM unveiled the presence of homologs in a wider number of phyla, as well as in a larger number of cyanobacterial species (Fig. S5 and S6).

### DNA-binding activity of NsrX and NsrM

**Fig 2:**
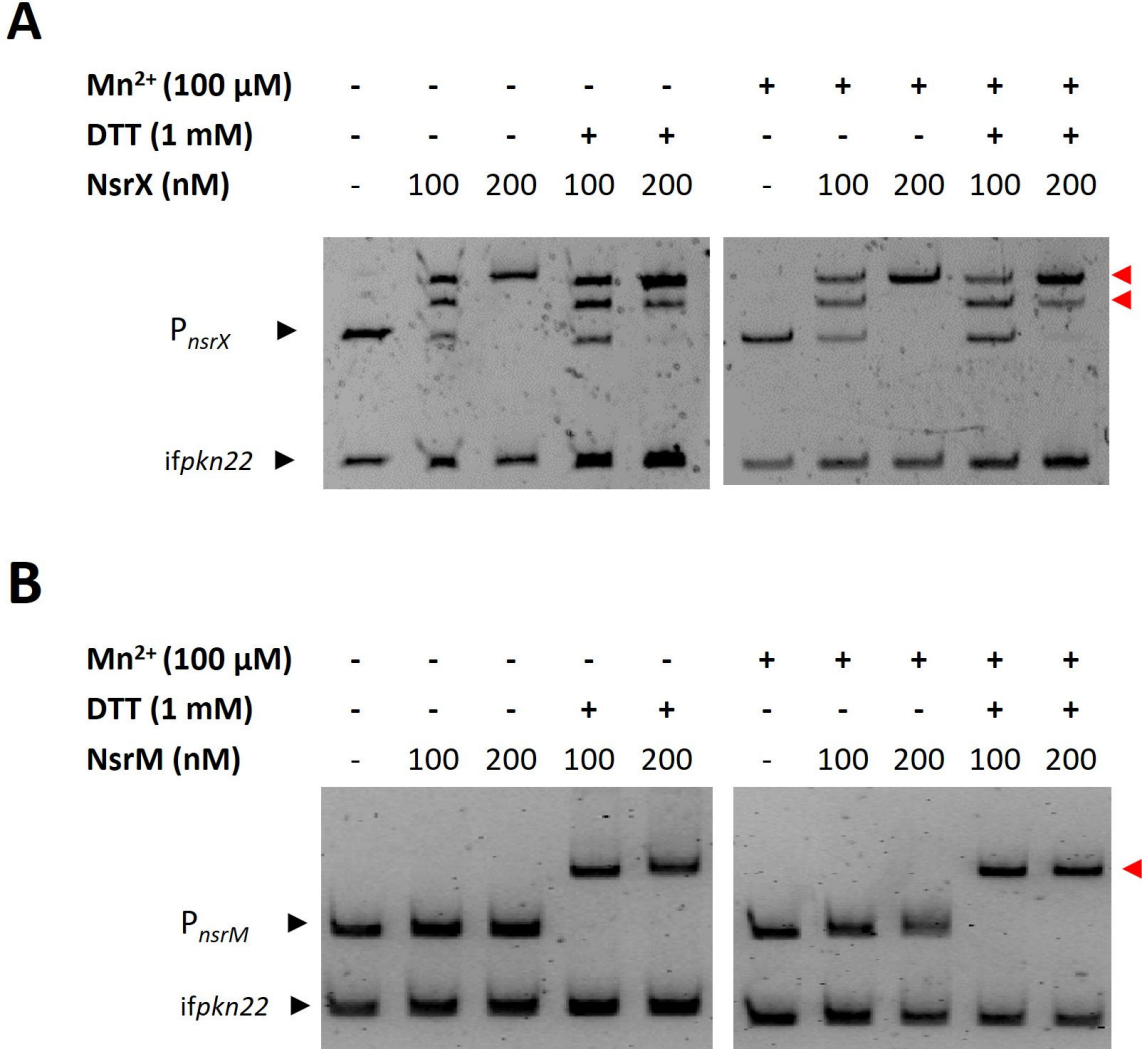
Analysis of DNA binding of NsrX (**A**) and NsrM (**B**) to their own promoter region. Recombinant NsrX and NsrM were incubated with their respective promoters in the presence or absence of 1 mM of DTT and in the presence or absence of 100 µM MnCl_2_. To analyze the effects of Mn on DNA binding, both gel and running buffer also included 100 µM MnCl_2_. Binding reactions were resolved by 6% PAGE. An internal fragment of the gene *pkn22* was used as non-specific competitor DNA.

Since a large number of transcriptional regulators are self-regulated, the ability of NsrX and NsrM to bind to their own promoter regions was assessed by electrophoretic mobility shift assays (EMSA) under different conditions (Fig. 2). Recombinant NsrX bound to its own promoter region with high affinity, regardless of metal and redox conditions. However, its lower-affinity binding to the *hepA*, *urtA*, *nblA,* and *nifHDK* promoters—which were further identified (see Fig. 4; Fig. S7)—was enhanced in the presence of Cu and Cd, with the largest effect observed with the addition of Mn^2+^, with the sole exception of the interaction with the *urtA* promoter (Fig. S7). In contrast, the interaction of NsrM with its own promoter region required reducing conditions (DTT) and was independent of the presence of divalent metals (Fig. 2B).

### NsrX and NsrM are potentially modulated by NtcA

**Fig 3:**
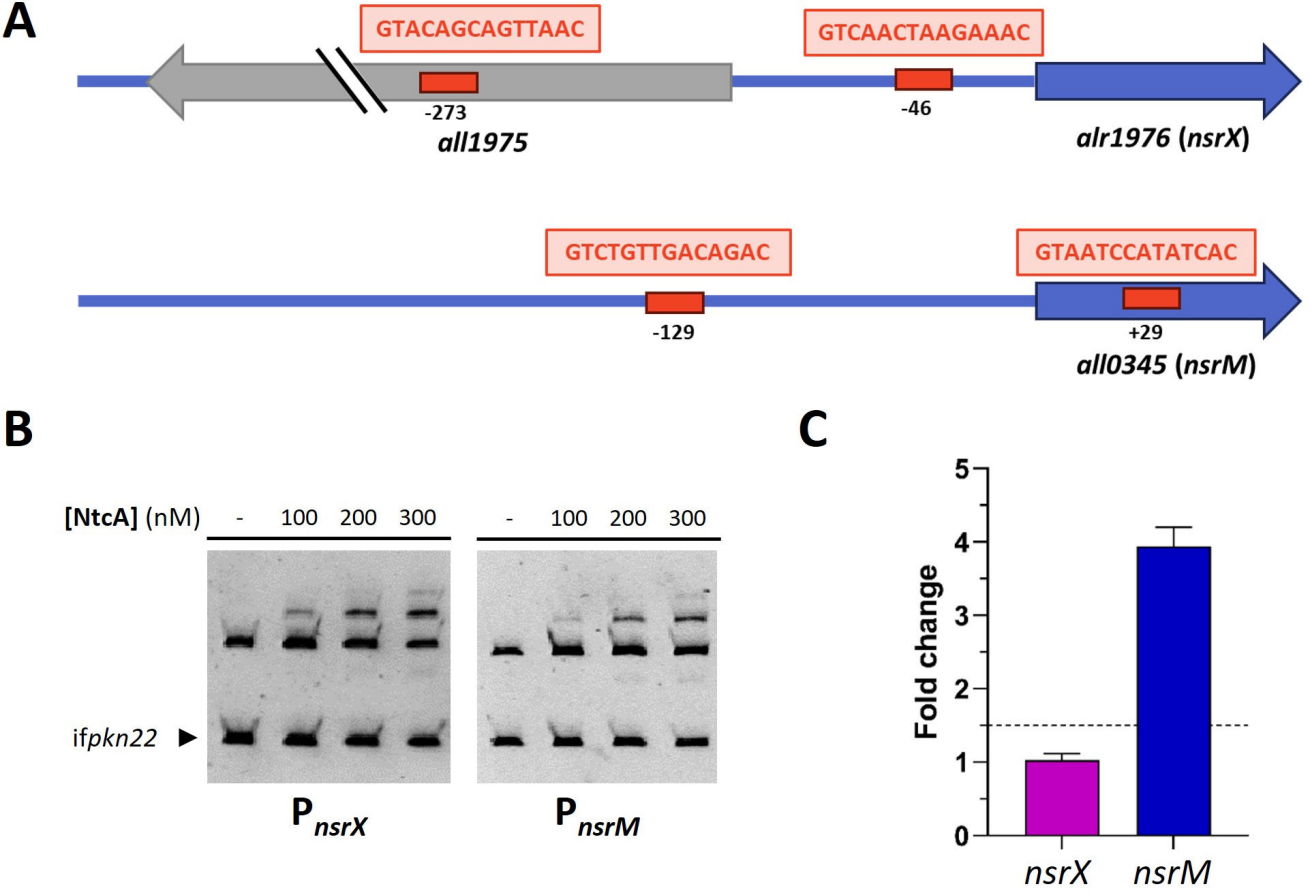
Modulation of *nsrX* and *nsrM* by NtcA. (**A**) Graphical representation of the putative NtcA boxes located in the promoter regions of *nsrX* and *nsrM*. The distances with respect to the start codons of the CDSs are indicated. (**B**) Electrophoretic mobility shift assays (EMSA) showing the ability of NtcA to bind *in vitro* to the promoter regions of *nsrX* and *nsrM*. DNA fragments free or mixed with increasing concentrations of recombinant NtcA (nM) were separated by 6% PAGE. An internal fragment of the gene *pkn22* was used as non-specific competitor DNA. (**C**) Levels of *nsrX* and *nsrM* mRNA in a *ntcA* deletion strain (CSE2) versus the wild-type strain. Relative real-time RT-PCR was used. Values are expressed as fold change and correspond to the average of three independent assays. The standard deviation is indicated.

Altered expression of *nsrX* and *nsrM* in a *furC*-overexpressing strain under nitrogen deficiency (12) suggests a link between these genes and nitrogen metabolism. Previous works highlighted that many members of the regulatory network of FUR proteins are co-regulated by the global nitrogen regulator NtcA (6, 13). However, the potential modulation of *nsrX* and *nsrM* by NtcA had not been previously explored. Since NtcA boxes were identified in the promoter regions of both regulators (Fig. 3A), we aimed to determine whether these genes could be modulated by NtcA. To address this question, we analyzed NtcA binding to the promoter regions of *nsrX* and *nsrM* using electrophoretic mobility shift assays (EMSA) and evaluated their differential expression in a *ntcA* deletion strain (CSE2) by real-time RT-PCR.

Figure 3B shows that NtcA binds to the promoter regions of *nsrX* and *nsrM*. The enhanced expression of *nsrM*, which increased almost fourfold in the Δ*ntcA* strain, suggests that NtcA could be acting as a transcriptional repressor of *nsrM* (Fig. 3C). However, despite the clear interaction observed *in vitro* between NtcA and the *nsrX* promoter, no significant differential expression of this regulator was detected in the Δ*ntcA* strain under the standard culture conditions tested.

### *In vitro* interaction of NsrX and NsrM with the promoter regions of genes involved in nitrogen metabolism and heterocyst differentiation

To further explore the potential relationship of NsrX and NsrM with the modulation of nitrogen metabolism, we investigated if these regulators directly interact with the regulatory regions of key nitrogen-related genes (Table 1). A selection encompassing promoters of genes involved in nitrogen assimilation and the sequential steps of heterocyst development, including initiation, pattern formation, commitment, envelope synthesis, and nitrogen fixation (3), was tested by EMSA in the presence of NsrX and NsrM under different conditions.

**TABLE 1:** Genes involved in nitrogen assimilation and heterocyst differentiation in *Anabaena* sp. strain PCC 7120 whose promoters were tested by EMSA with the transcriptional regulators NsrX and NsrM^*a*^

	NsrX	NsrM		NsrX	NsrM
Nitrogen Assimilation			Heterocyst envelope		
*nir* operon		**+**	*hepA*	**+**	
*urtABCDE*	**+**		*hepB*		**+**
*glnA*	**+**		*hepC*		
*nblA*	**+**	**+**	*hepK*	**+**	**+**
Initiation of heterocyst differentiation			*hglT*		
*ntcA*			*hgdC*	**+**	
*hetR*	**+**	**+**	*hgdD*		
*nrrA*		**+**	*all0809-07*	**+**	
*hanA*			*alr3646-49*		**+**
*sigC*		**+**	*alr4973-75*		
*sigE*			*henR*		
*sigG*			*devR*		
Pattern formation			*devH*		
*asr1734*		**+**	*hepS*		
*patA*		**+**	*hepN*		**+**
*patB*			Nitrogen fixation and O_2_ reduction		
*patS*	**+**		*nifHDK*	**+**	
*patX*		**+**	*xisHI*		
*hetC*		**+**	*rbrA*		
*hetN*					
*hetL*					
Commitment					
*hetP*					
*hetZ*	**+**				

^*a*^ Genes for which binding of NsrX and NsrM to the promoter region was observed are indicated as +.

**Fig 4:**
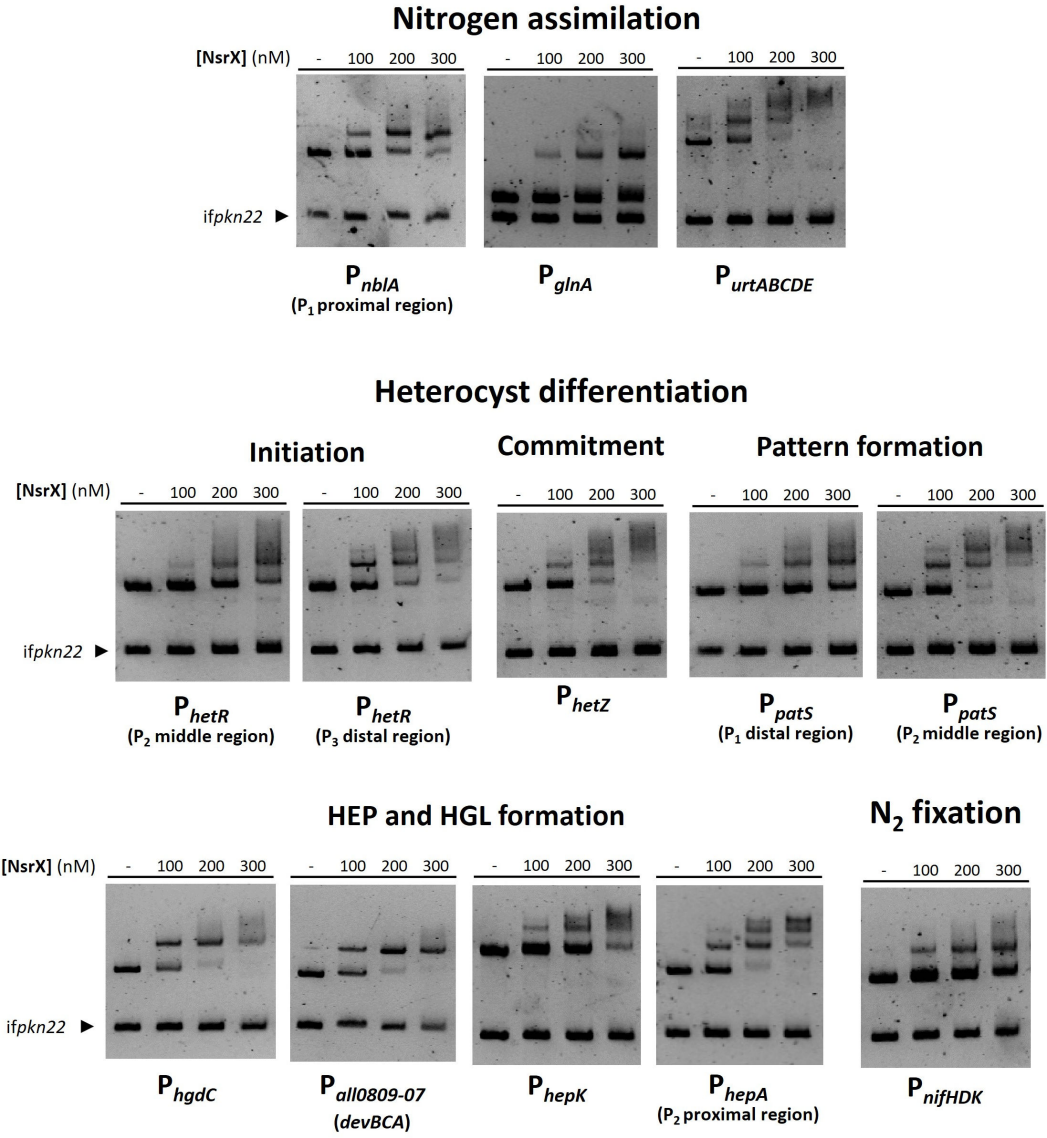
EMSA assays showing the ability of NsrX to bind *in vitro* to the promoter regions of genes involved in nitrogen metabolism and heterocyst differentiation. DNA fragments free or mixed with increasing concentrations of recombinant NsrX (nM) were separated by 6% PAGE. Incubation was done in the presence of 100 µM MnCl_2_, and both gel and running buffer also included 100 µM MnCl_2_. An internal fragment of the gene *pkn22* was used as non-specific competitor DNA. Proximal region of *nblA* promoter spans from −211 to +3 bp relative to the translation start codon. Middle and distal regions of the *hetR* promoter span from −629 to −270 bp and from −949 to −629 bp relative to the translation start codon, respectively. Middle and distal regions of the *patS* promoter span from −610 to −310 bp and from −925 to −610 bp relative to the translation start codon, respectively. Proximal region of the *hepA* promoter spans from −268 to +51 bp relative to the translation start codon.

The XRE-like regulator NsrX binds to the promoter region of genes crucial for nitrogen assimilation (*nblA*, *glnA*, *urtABCDE*), initiation of heterocyst differentiation (*hetR*), commitment (*hetZ*), pattern formation (*patS*), synthesis of the envelope (*hgdC*, *all0809-07* (*devBCA*), *hepK, hepA*) and nitrogen fixation (*nifHDK*) (Fig. 4). In contrast to the metal-independent high-affinity interaction of NsrX with its cognate promoter, its binding to other promoters, such as P*_urtA_* and P*_hepA_*, occurred with lower affinity and was enhanced by the presence of Cu and Cd, with Mn exhibiting the largest effect (Fig. S7). Overall, the presence of Mn improved NsrX interaction with most of its targets (for some examples, see Fig. S7).

**Fig 5:**
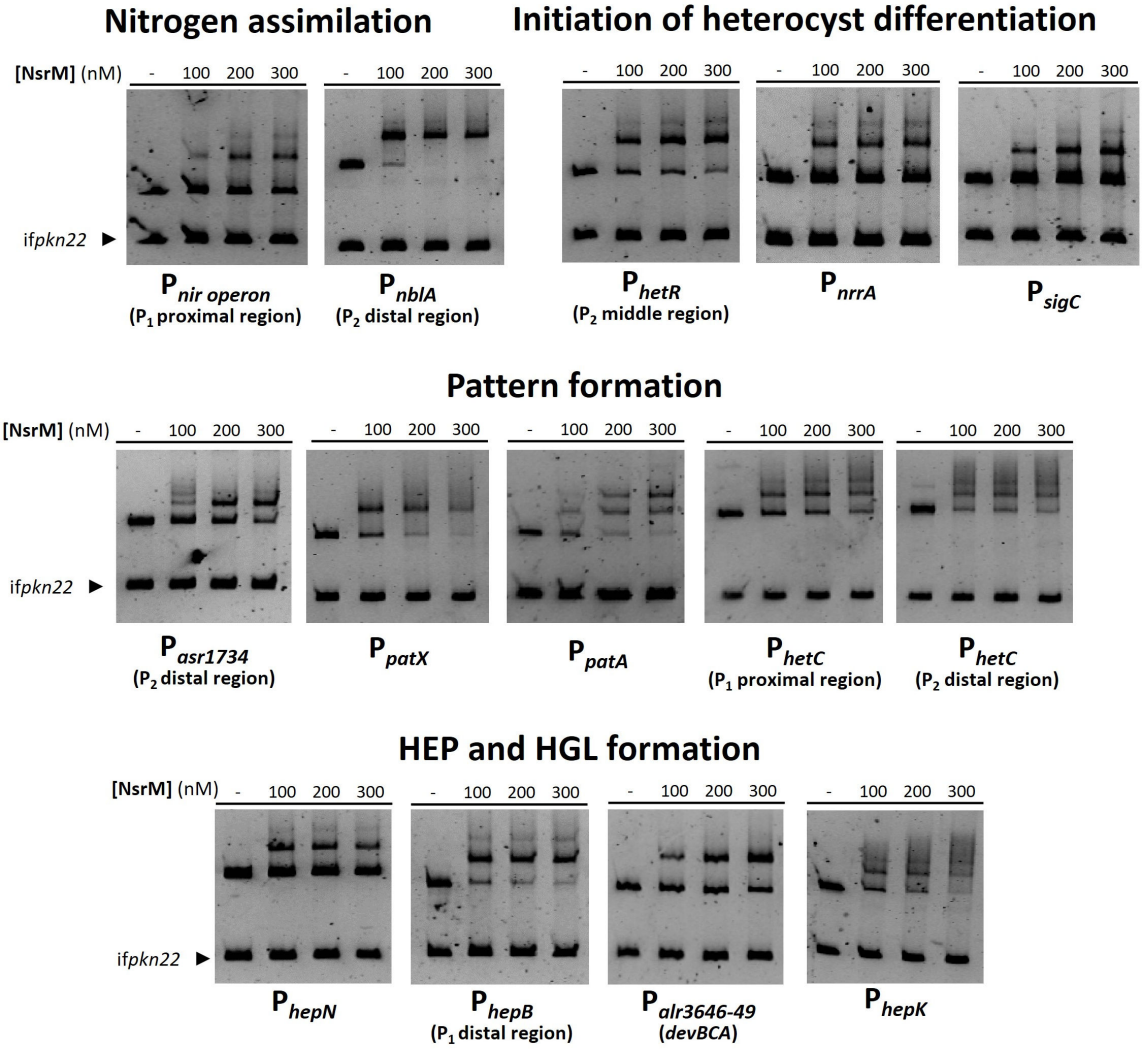
EMSA assays show the ability of NsrM to bind *in vitro* to the promoter regions of genes involved in nitrogen metabolism and heterocyst differentiation. DNA fragments free or mixed with increasing concentrations of recombinant NsrM (nM) were separated by 6% PAGE. Incubation was done in the presence of 1 mM DTT. An internal fragment of the gene *pkn22* was used as non-specific competitor DNA. The distal region of the *nblA* promoter spans from −496 to –191 bp relative to the translation start codon. Middle regions of the *hetR* promoter span from −629 to −270 bp relative to the translation start codon. The distal region of the *asr1734* promoter spans from −494 to −171 bp relative to the translation start codon. Proximal and distal regions of the *hetC* promoter span from −411 to 0 bp and from −874 to −411 bp relative to the translation start codon, respectively. The distal region of the *hepB* promoter spans from −694 to −322 bp relative to the translation start codon.

The MerR-like regulator NsrM specifically binds to the promoter region of genes involved in nitrogen assimilation (*nir* operon, *nblA*), initiation of heterocyst differentiation (*hetR*, *nrrA*, *sigC*), pattern formation (*asr1734*, *patX*, *patA*, *hetC*) and synthesis of the envelope (*alr3646-49*/*devBCA*, *hepB*, *hepN*) (Fig. 5).

### Differential expression of NsrX and NsrM targets in Δ*nsrX* and Δ*nsrM* mutants

**Fig 6:**
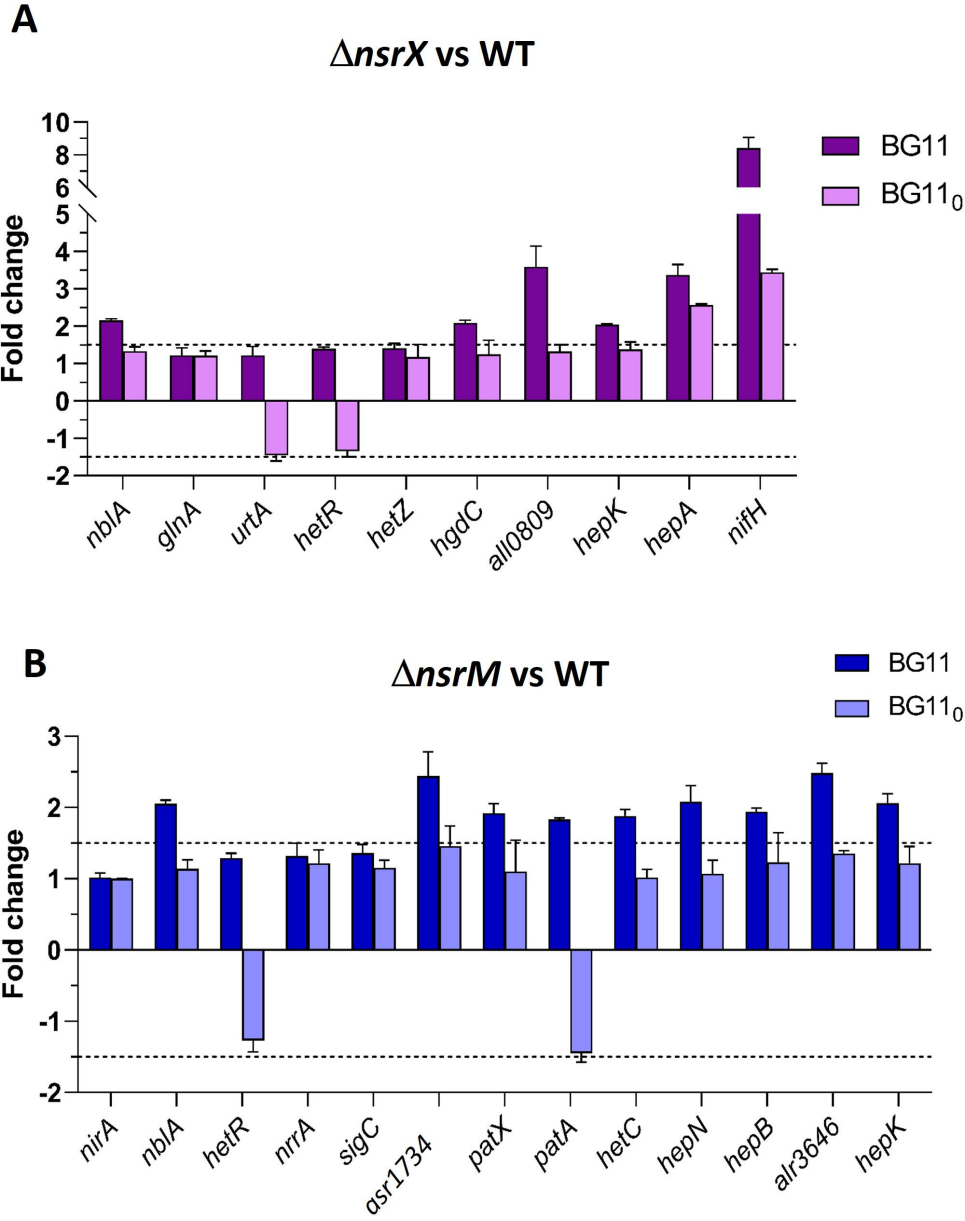
Influence of *nsrX* deletion (**A**) and *nsrM* deletion (**B**) on the mRNA levels of their target genes under standard conditions (BG11) and after 48 h of nitrogen deficiency (BG11_0_). Relative real-time RT-PCR was used. Values are expressed as fold change (Δ*nsrX* vs WT or Δ*nsrM* vs *Anabaena* WT) and correspond to the average of three biological and three technical replicates. The standard deviation is indicated.

To better understand the effects of NsrX and NsrM on the expression of their newly identified target genes, real-time RT-PCR analyses were performed. Differential expression of these targets was analyzed in the corresponding deletion strains (Δ*nsrX* or *ΔnsrM*) versus the wild-type *Anabaena* sp. strain PCC 7120 under standard conditions (BG11) and after 48 h of nitrogen deficiency (BG11_0_) (Fig. 6). Due to the short length of the transcript (54 nt), the expression of *patS* could not be determined by real-time RT-PCR.

Under standard conditions, six of the 10 identified NsrX targets were differentially expressed in the △*nsrX* strain. The expression of *nifH*, *hepA*, and *all0809* underwent substantial changes in the △*nsrX* mutant, suggesting their direct regulation by NsrX (Fig. 6A). In contrast, the fold changes for *nblA*, *hgdC*, and *hepK* were more modest. No significant changes were observed in the expression of *glnA*, *urtABCDE*, *hetR*, and *hetZ* under the conditions tested. In the case of NsrM, most of the targets that resulted positive in EMSA analysis (nine of the thirteen promoters identified) exhibited altered expression levels in △*nsrM* cells (Fig. 6B). Among them, the most relevant changes corresponded to *asr1734* and *hepB*. A weaker differential expression was found for *nblA*, *patX*, *patA*, *hetC*, *hepN*, *alr3646*, and *hepK*, while no changes were observed in *nirA*, *hetR*, *nrrA*, and *sigC*.

Interestingly, all differentially expressed genes showed an upregulation in the corresponding deletion strain versus the wild-type *Anabaena*, but in all cases, this induction was lowered or even was not present under nitrogen deficiency. Furthermore, except for *nifHDK* and *devBCA* in the *Δalr1976* strain, which showed upregulations of 8.4-fold and 3.6-fold, respectively, the relevant genes in nitrogen metabolism, including *nblA*, *hgdC*, *hepK*, and *hepA*, showed moderate inductions (~2-fold).

In addition, changes in the expression of NsrX and NsrM targets after 48 h of nitrogen deficiency (BG11_0_) were analyzed in the corresponding deletion strains and the WT *Anabaena* (Fig. S8). Overall, genes involved in nitrogen assimilation and heterocyst differentiation were induced after 48 h of nitrogen deficiency in the WT strain, but this induction was lowered in the corresponding deletion strain, demonstrating the contribution of both regulators to achieve a fully efficient cell response and adaptation to nitrogen deprivation.

### Heterocyst differentiation in Δ*nsrX* and Δ*nsrM* strains

**Fig 7:**
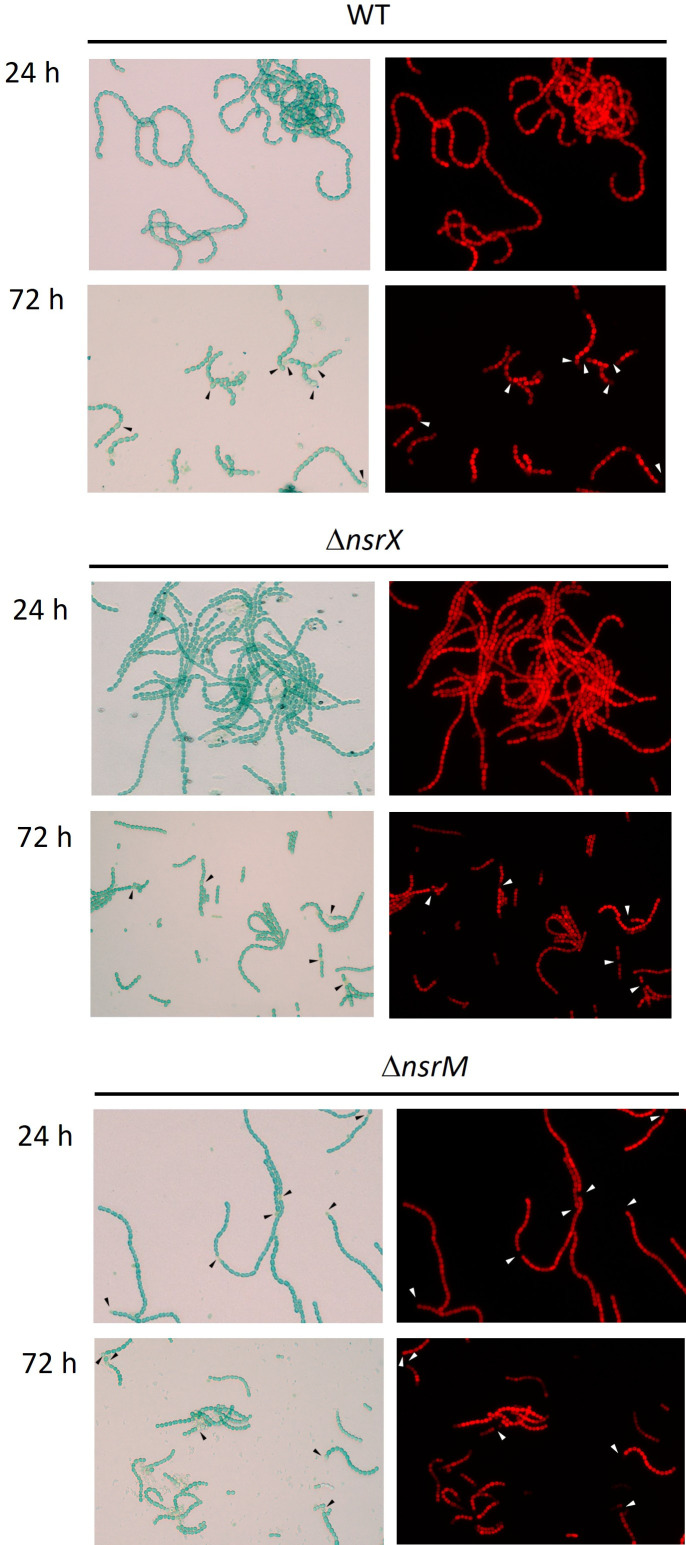
Bright-field micrographs of *Anabaena* sp. strain PCC7120, Δ*nsrX,* and Δ*nsrM* strains after 24 and 72 h of nitrogen step-down. Cells were observed under bright field and fluorescence microscopy to observe phycobiliprotein intrinsic fluorescence. Images are representative of at least 10 different fields of three biological replicates. Heterocysts are marked with arrowheads.

Analysis of heterocyst frequency and timing of differentiation in wild-type *Anabaena* (WT), Δ*nsrX,* and Δ*nsrM* strains showed no significant alterations on heterocyst morphology, heterocyst patterning, or frequency (Fig. 7; Table 2). Alcian Blue staining revealed that all three strains are capable of developing mature heterocysts with a functional polysaccharide layer. However, we found that the Δ*nsrM* strain exhibited earlier heterocyst formation, with Alcian Blue-stained heterocysts observed at 24 h, unlike the WT and Δ*nsrX* strains, which did not show mature heterocysts at this time point (Fig. S9).

**TABLE 2:** Heterocyst formation in *Anabaena* strains after nitrogen step-down^*a*^

Strain	WT	Δ*nsrX*	Δ*nsrM*
% heterocysts	5.24 ± 0.04	4.99 ± 0.24^ns^	5.42 ± 0.07^ns^
Time elapsed until heterocyst formation	48 h	48 h	24 h

^*a*^ Percentages of heterocysts and the time required for their formation were determined in *Anabaena* sp. strain PCC7120, Δ*nsrX*, and Δ*nsrM* cultures after 72 h of combined nitrogen step-down. Measurements were made using both fluorescence analysis and Alcian Blue staining. All cell counts were performed on three independent biological replicates.

## DISCUSSION

While bacterial master regulators have been extensively studied, the roles of most accessory, yet important, transcriptional regulators in the modulation of regulatory networks remain unknown. This was the case of *alr1976* (*nsrX*) and *all0345* (*nsrM*), which are modulated by FurC (PerR). Based on bioinformatics analyses, *nsrX* and *nsrM* encode transcriptional regulators belonging to the XRE and MerR families, respectively. Previous works found that both regulators were direct targets of FurC (13) and were differentially expressed in a *furC*-overexpressing strain under nitrogen deficiency (12). Our findings demonstrate that *nsrM* is under the control of NtcA, while the regulation of *nsrX* is uncertain. Notably, *nsrX* and *nsrM* contain NtcA binding boxes in their promoter regions, and EMSA assays showed that there is a clear interaction with both promoters. Although no significant differential expression for *nsrX* was observed in the Δ*ntcA* strain under standard culture conditions, a similar situation was observed with other regulatory genes belonging to the FUR regulatory network, for which direct binding of NtcA to their promoter region has been described (13). This could be attributed to the fact that the final expression of these genes is also influenced by the modulation exerted by other regulators and could integrate different cellular signals. For example, in the case of *pkn22*, it was found that NtcA could bind to the promoter of this gene, but differential expression in the Δ*ntcA* strain only occurred under oxidative stress (10). Furthermore, we cannot discard that NsrX could play an indirect modulatory role in the nitrogen regulation network, potentially influencing the expression of the genes reported here, without being a direct NtcA target itself.

NsrX and NsrM bind to the promoter regions of genes involved in nitrogen assimilation and heterocyst differentiation, and deletion of *nsrX* and *nsrM* results in altered expression of some of their potential target genes. However, given the modest transcriptional changes exhibited by most of the identified promoters, the *in vivo* relevance of these interactions warrants further investigation. Nevertheless, significant alterations in the differential expression of *nifH*, *hepA*, and *devB* were observed in the Δ*nsrX* cells, and of *asr1734* and *hepB* in Δ*nsrM* cells, unveiling the contribution of NsrX and NsrM to the transcriptional control of cellular responses to nitrogen deficiency.

One important question is whether these regulators act as activators or repressors. According to transcriptional changes, both NsrX and NsrM seem to work as transcriptional repressors, since the expression of their target genes increases in the corresponding deletion mutant. This is in agreement with the traditional mechanism of action of XRE regulators that typically act as repressors of gene expression (21), while MerR regulators exhibit a dual functionality, acting as both transcriptional activators and repressors (17, 22). It is noticeable that MerR regulators remain bound to DNA both in the absence and presence of their cognate signals, which can include oxidative stress, metals, or organic molecules (16). Transcriptional activation occurs only upon signal binding, which induces a conformational change in MerR that alters DNA conformation within the promoter region and drives gene activation (17). Indeed, it has been proven that the MerR regulator, which controls mercury resistance genes, represses target gene expression in the absence of Hg(II) by hindering the binding of RNA polymerase to the promoter region (23). Consequently, under standard culture conditions used in this work, NsrM might lack its specific activation signal, thereby repressing the expression of its target genes.

Elucidating the regulatory mechanisms governing NsrX and NsrM activity is crucial for fully understanding their roles in *Anabaena* sp. strain PCC 7120. In this work, we have demonstrated that NsrX binding to DNA is generally enhanced by the presence of Mn^2+^, and to a lesser extent by Cu^2+^ and Cd^2+^, whereas NsrM DNA binding requires reducing conditions. Consequently, both regulators would be able to integrate cellular signals, such as metal availability or redox status, to control the expression of genes involved in responses to nitrogen deficiency. Indeed, there are increasing evidences revealing the interplay between these three cellular processes (12, 24–27). However, we cannot rule out the possibility that additional signals may also influence NsrX and NsrM DNA-binding activity.

In the case of NsrX, bioinformatic studies revealed that it is potentially co-transcribed with a gene encoding Alr1977, a Zn-metalloprotease homolog with an N-terminal IrrE domain. Although the role and targets of this protease in *Anabaena* sp. strain PCC7120 are unknown, the fact that this tandem is conserved among cyanobacteria suggests that Alr1977 might modulate NsrX by proteolysis. Indeed, previous works described that XRE-IrrE protease pairs are found in many bacteria and proposed two different molecular mechanisms for stress-induced gene de-repression: protease-mediated cleavage or inhibition of oligomerization without cleavage of the XRE repressor (28). By analogy, Alr1977 could play a similar role in *Anabaena*, regulating NsrX activity in response to environmental or cellular signals. Further studies are needed to determine whether Alr1977 directly cleaves NsrX, how this process is modulated by specific signals, and whether this potential regulatory mechanism is connected to nitrogen metabolism and heterocyst differentiation.

With respect to NsrM, bioinformatic predictions revealed the presence of an AraC-E effector-binding domain, which is likely to be involved in the binding of small molecules that affect its activity. For example, the BmrR regulator from *Bacillus subtilis*, which contains a similar AraC-E effector-binding domain, is able to control gene expression in response to drug-like compounds (29). This means that NsrM is likely to modulate gene expression not only in response to redox status but also in response to other unknown signals. Additional work should determine which signal(s) are sensed by this regulator, how these signals are connected to the response to nitrogen deficiency, and what their effects are on gene expression.

Our findings suggest that both NsrX and NsrM contribute to the modulation of genes involved in nitrogen assimilation and heterocyst differentiation. This is supported by our observations that they bind to the promoter region of these genes, and that differential expression was observed in their corresponding deletion strains. Albeit in many cases, fold changes were small, most NsrX and NsrM targets were upregulated in the corresponding deletion strain under standard culture conditions (BG11). However, this was not the case under nitrogen deprivation (BG11_0_), when almost all target genes were not differentially expressed. These findings indicate that both regulators could be repressing genes involved in nitrogen deficiency responses under standard conditions, while the lack of upregulation under nitrogen deprivation suggests that they might allow the de-repression of these targets in these conditions. The mechanisms mediating this potential de-repression are unknown, but based on bioinformatic analyses, we hypothesize that it could take place by means of Alr1977-mediated proteolysis of NsrX or by the binding of some molecules to the AraC-E effector-binding domain of NsrM.

Another possible explanation for this absence of upregulation of NsrX and NsrM targets under nitrogen deficiency could be that both regulators are expressed at lower levels in these conditions, resulting in minimal impact upon deletion. Indeed, it was found that *nsrX* and *nsrM* expression is lowered after 21 h of nitrogen deficiency (30). These data also indicate that during nitrogen deficiency, *nsrX* expression is downregulated, while *nsrM* undergoes a transient induction followed by a downregulation (Fig. S10). At the same time, a strong induction of *ntcA* and a more modest induction of *furC* are observed, a fact that aligns with a model in which FurC activates *nsrM* and represses *nsrX* during heterocyst differentiation, while the later downregulation of *nsrM* may be attributed to NtcA repression.

Previous studies found that changes in heterocyst patterning and frequency, as well as formation of heterocyst under nitrogen sufficiency, are mainly observed when expression of important regulators such as *hetR* or genes involved in commitment, such as *hetZ* and *hetP,* was altered at least ±4-fold (31, 32). Consequently, either the absence or moderate fold changes in most of the heterocyst formation genes in the *nsrX* and *nsrM* strains could explain why we did not observe any significant changes in heterocyst morphology, patterning, or frequency in these mutants. Furthermore, despite the upregulation of heterocyst formation genes under standard culture conditions, no heterocyst formation was observed in the presence of NO_3_^-^. This is consistent with the occurrence of hierarchically superior transcriptional regulators that are actively repressing heterocyst differentiation. The only phenotypic effect found was that in the Δ*nsrM* mutant, heterocysts were formed 24 h earlier than in the wild type. This suggests that NsrM, while not solely responsible, may contribute to repress heterocyst differentiation under nitrogen sufficiency. This role has previously been reported for other regulators such as CalA, which acts as a safety device to prevent heterocyst differentiation (33).

**Fig 8:**
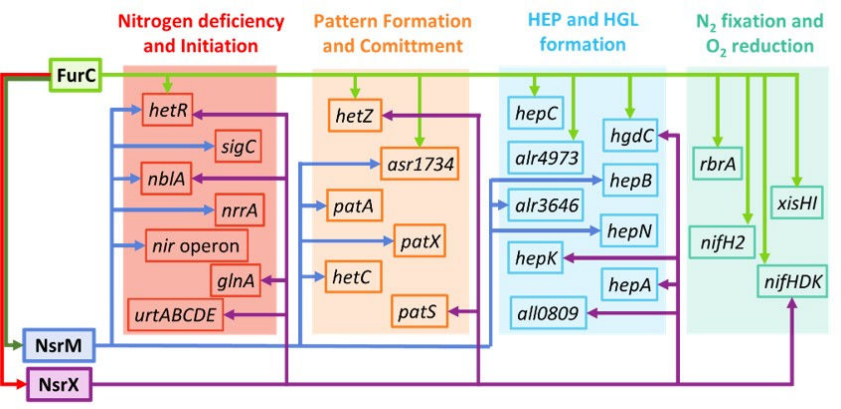
A scheme showing the coordinated regulation of genes involved in nitrogen assimilation and heterocyst differentiation by FurC and its two targets NsrX and NsrM. Arrowheads denote target genes of the regulons of FurC (light green line), All0345/NsrM (blue line), and Alr1976/NsrX (purple color). The red arrow indicates downregulation of NsrX by FurC, while the dark green represents activation of NsrM by FurC.

Complementary data (Fig. S8) indicate that after 48 h of nitrogen step-down, the induction of NsrX and NsrM target genes is weaker in their respective deletion mutants, but in almost all cases, it still takes place. This was expected, as heterocyst formation is not affected in these strains, and these regulators are not the only modulators of heterocyst differentiation. Therefore, although not essential for heterocyst formation, NsrX and NsrM possibly play a role in integrating signals such as metal availability or redox status within the complex regulatory cascade governing heterocyst differentiation, allowing a more precise modulation of gene expression. Since both regulators are under control of FurC and likely of NtcA, it is feasible that NsrX and NsrM enable FurC and NtcA to finely tune expression during heterocyst differentiation. Interestingly, NsrX and NsrM share several gene targets with FurC (i.e., *hetR*, *hetZ*, *asr1734*, *hgdC*, and *nifHDK*) (12, 34). This overlap suggests that the interplay of these regulators may constitute a mechanism that would enable FurC to exert a more precise control of the expression of these genes (Fig. 8). However, other genes, such as *patS*, *patA*, *patX*, *hepA*, *hepB*, *hepK*, *hepN*, and *hetC,* are not direct targets of FurC. Therefore, NsrX and NsrM would expand the FurC regulon. This could also explain why genes, such as *sigC*, *patA*, and *alr3646*, exhibit transcriptional changes in the FurC overexpression strain transcriptome despite not being direct FurC targets (12).

In summary, this study establishes NsrX and NsrM as transcriptional regulators that likely contribute to the modulation of the genetic responses to nitrogen deficiency in *Anabaena* sp. strain PCC 7120. These regulators could integrate various environmental signals, such as metal availability and redox status, into the genetic control of these processes. The subtle phenotypes observed in the Δ*nsrX* and Δ*nsrM* mutants suggest that these secondary regulators exert their influence through fine-tuning or buffering effects, which may become critical under specific physiological conditions or in conjunction with other regulatory pathways. While the deletion of *nsrX* or *nsrM* did not yield a pronounced phenotype under the conditions tested, future research under different environmental or stress scenarios is necessary to fully assess their impact in the cyanobacteria. The putative roles of NsrX and NsrM in fine-tuning the expression of genes involved in nitrogen assimilation and heterocyst formation, together with their potential to expand the FurC regulon, would add an additional layer to the network that coordinates the transcriptional responses necessary for the efficient adaptation to nitrogen deprivation. Future studies will be essential to uncover the precise mechanisms through which NsrX and NsrM modulate their target genes and integrate other potential regulatory signals into the complex heterocyst differentiation cascade.

## MATERIALS AND METHODS

### Protein purification

Recombinant His-tagged NsrX, NsrM, and NtcA were obtained as recombinant proteins from *Escherichia coli* BL21(DE3) cells. Cloning and purification procedures for NsrX and NsrM are detailed in Supplementary information File S1. NtcA was purified as previously described by I. Álvarez-Escribano et al. (35) used *E. coli* BL21 (DE3) cells harboring plasmid pSAM334, which was kindly provided by Dr. Antonia Herrero (CSIC, Spain).

### Electrophoretic mobility shift assays (EMSA)

NtcA binding to *nsrX* and *nsrM* promoters, as well as NsrX and NsrM binding to the promoter regions of their target genes, was analyzed by electrophoretic mobility shift assays (EMSA). Promoter regions used in the analyses consisted of 300–400 bp DNA fragments upstream of the ATG start codon and were obtained by PCR using *Anabaena* sp. strain PCC 7120 genome as template, with primers listed in Table S1. In the case of genes whose promoter regions were larger than 400 bp, the promoter region was analyzed in several fragments of 300–400 bp.

EMSA reactions were performed by mixing purified NsrX, NsrM, or NtcA in a final volume of 20 µL with 50 ng of DNA promoters in a binding buffer containing 10 mM Bis Tris–HCl (pH 7.5), 40  mM KCl, 0.1 mg/mL BSA, and 5% (vol/vol) glycerol. When indicated, 1 mM of 1,4-dithiothreitol (DTT) and/or 100 µM of the specified divalent metals were added to the binding mix. To probe specific binding, a competitor DNA was used, which consisted of 50-100 ng of a 150 bp internal fragment of gene *pkn22* (if*pkn22*). The resulting mixture was incubated for 30  min at room temperature, mixed with 3 µL 6 × loading buffer (30  mM Bis-Tris pH 8, 30% glycerol, and 0.05% bromophenol blue), and loaded into a non-denaturing 6% polyacrylamide gel. Electrophoresis was run at 4°C under a voltage of 90 V for approximately 110  min in 50 mM Tris-HCl 380 mM Glycine, pH 8.5. In the case of assays done in the presence of metals, both gel and running buffer included 100 µM of either MnCl_2_, CdCl_2_, CoCl_2_, ZnSO_4_, and CuSO_4_. Gels were stained with SYBR Safe (Invitrogen) and visualized in a GelDoc 2000 device (Bio-Rad).

### Construction of *nsrX* and *nsrM* deletion strains in *Anabaena* sp. strain PCC 7120

The *alr1976* and *all0345* deletion strains (∆*nsrX* and ∆*nsrM*) were constructed using CRISPR-Cpf1 genome editing, following cloning methods and genome editing procedures reported previously (36). Detailed procedures are described in Supplementary information File S2.

### Cell cultures and RNA extraction

Cyanobacterial strains used in this work included the wild-type *Anabaena* sp. strain PCC 7120 and the *nsrX*, *nsrM*, and *ntcA* deletion strains (∆*nsrX* , ∆*nsrM* and CSE2). ∆*nsrX* and ∆*nsrM* strains were obtained in this work, whereas the CSE2 strain is an insertional mutant of the *ntcA* gene (37). Cultures were grown photoautotrophically in either BG-11 medium or BG-11_0_ (BG11 without NaNO_3_) for nitrogen-deprived conditions (38). The only exception was the CSE2 strain, which was cultivated in BG11_0_ supplemented with 6 mM NH_4_Cl and 12 mM TES-NaOH buffer (pH 7.5), 2  µg mL^−1^ streptomycin, and 2  µg mL^−1^ spectinomycin. All experiments were conducted using three biological and two technical replicates. Cultures were set up by diluting late exponential-phase cells to a starting OD_750_ of 0.4 in a final volume of 100  mL. Cell cultures were maintained at 28°C in 250 mL Erlenmeyer flasks on an orbital shaker at 120  rpm under continuous illumination of 30 μE m^−2^ s^−1^. In the case of nitrate-deprived samples, cells grown in standard BG-11 were collected by centrifugation, washed three times with BG-11_0_, and resuspended in BG-11_0_ to the final optical density. RNA was extracted and purified from samples of 25 mL of each culture following previously described methods (12).

### Real-time RT-PCR

The pool of cDNAs was synthesized by reverse transcription of 2  µg of total RNA using SuperScript retrotranscriptase (Invitrogen) following the manufacturer’s conditions. Real-time PCR was performed using the QuantStudio 5 system (Applied Biosystems). Each reaction was set up by mixing 12.5  µL of SYBR Green PCR Master Mix with 0.4  µL of 25  µM primer mixture and 10  ng of cDNA template in a final volume of 30  µL with nuclease-free water (Ambion); additional water was added instead of cDNA for negative controls. Amplification was performed at 60°C for 40 cycles. The sequences of specific primers of selected genes were designed with Primer Express 3 (Thermofisher) and are listed in Table S1. Transcript levels of target genes were normalized to those of the housekeeping gene *rnpB* (39). Relative quantification and expression fold changes were calculated according to the comparative Ct method (ΔΔCt method) (40), with a fold change threshold set at ≥1.5 or ≤−1.5.

### Heterocyst visualization

Bright-field examinations of *Anabaena* sp. strain PCC 7120, ∆*nsrX,* and ∆*nsrM* were performed after 24, 48, and 72  h of culture in BG11 and BG11_0_. Forty microliters of culture were immobilized in BG11 agar for nitrogen-sufficient conditions and in BG11_0_ agar for nitrogen-deprived conditions. Samples were placed on an inverted sample holder and visualized using an Olympus IX81 microscope under a 40× objective. Heterocyst percentage values at 72 h under nitrogen deficiency were obtained through a combination of manual and automated counting using image processing software ImageJ as described previously (9). The resulting heterocyst percentages from three independent assays were used to obtain a mean value for each strain, with a minimum of 1,000 estimated cells counted in total per strain. For staining of heterocyst polysaccharide layers, cell suspensions were mixed (5:1) with a 1% (wt/vol) Alcian Blue 8GX (Panreac) solution in water before its immobilization in agar.

### Bioinformatic tools

Protein sequences of Alr1976/NsrX and All0345/NsrM were retrieved from Uniprot (https://www.uniprot.org/). Domain and motif prediction was performed using Prosite (https://prosite.expasy.org/), Interpro (https://www.ebi.ac.uk/interpro/), and SMART (https://smart.embl-heidelberg.de/). Secondary structure was predicted using PSIPred (http://bioinf.cs.ucl.ac.uk/psipred/), while tertiary structure was predicted using AlphaFold (https://alphafold.ebi.ac.uk/). Prediction of dimers was done using Swiss-Model (https://swissmodel.expasy.org/), and superimposition with protein-DNA complexes was performed using Chimera MatchMaker tool (41). Structures were visualized using Chimera (42). Searches for NsrX and NsrM homologs in other prokaryotes, as well as conservation of the XRE–protease tandem, were performed using STRING v.11.5 database (https://string-db.org/).

## SUPPLEMENTAL MATERIAL

Supplemental figures spectrum.02311-25-s0001.pdf Figures S1 to S10.

Files S1 and S2 spectrum.02311-25-s0002.docx Supplemental methods for NsrX and NsrM purification and for construction of nsrM and nsrX deletion strains in *Anabaena* sp. PCC 7120.

Table S1 spectrum.02311-25-s0003.docx Oligonucleotides used in this study.

ASM does not own the copyrights to Supplemental Material that may be linked to, or accessed through, an article. The authors have granted ASM a non-exclusive, world-wide license to publish the Supplemental Material files. Please contact the corresponding author directly for reuse.
