# Supplementary material for: NsrM (All0345) and NsrX (Alr1976), two FurC (PerR)-targeted transcriptional regulators, modulate nitrogen metabolism and heterocyst differentiation genes in the cyanobacterium Anabaena sp. strain PCC 7120
Source: Microbiol Spectr. 2025 Oct 13;13(11):e02311-25. doi: 10.1128/spectrum.02311-25 (PMC12584617; doi:10.1128/spectrum.02311-25)
Supplement: Supplemental figures — Figures S1 to S10. [file spectrum.02311-25-s0001.pdf]

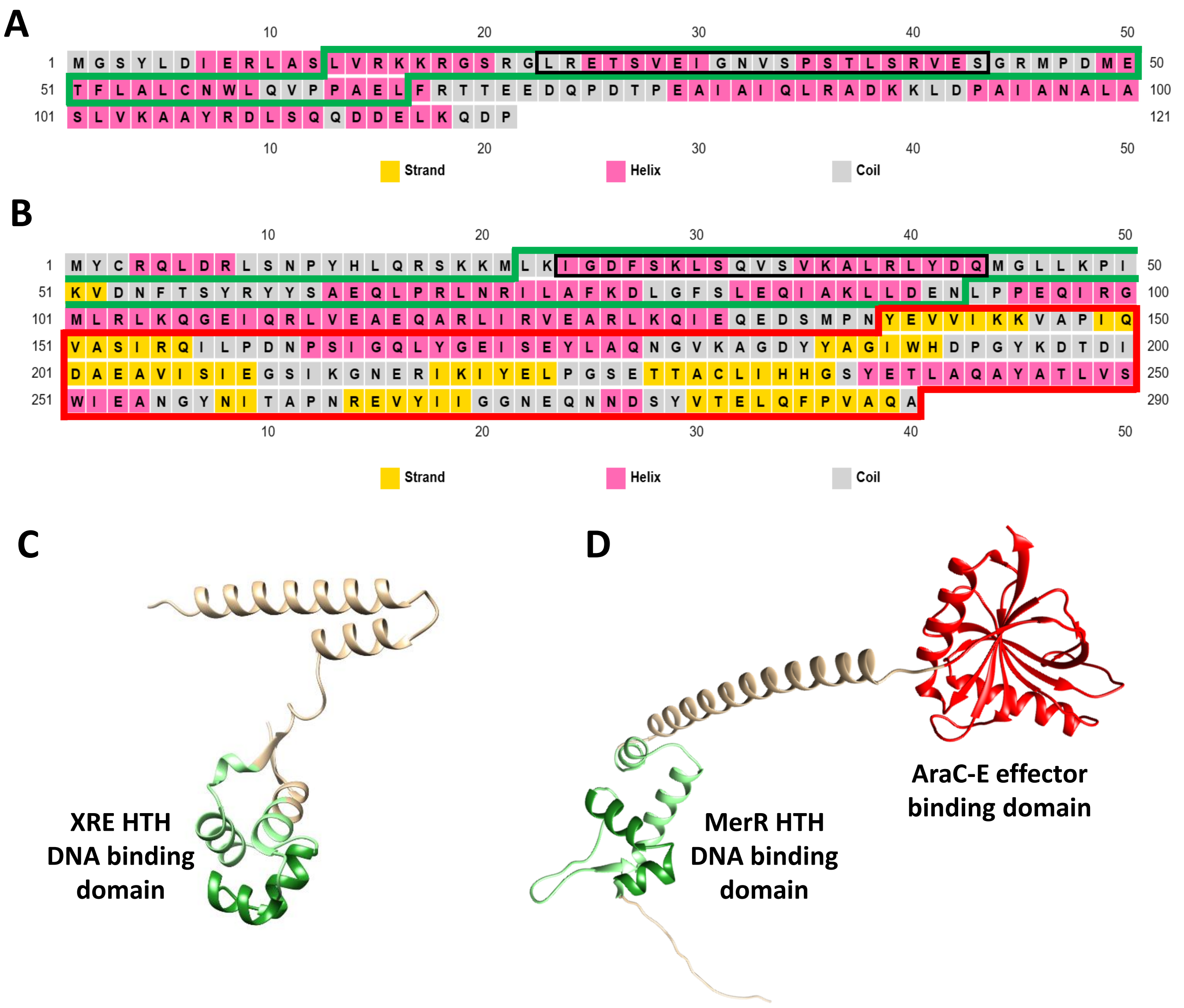

Figure S1. Secondary and tertiary structure predictions of Alr1976 (NsrX) and All0345 (NsrM) monomers. A. PSIPred secondary structure predictions of Alr1976 **(B)** and All0345. **C.** Tertiary structure prediction of Alr1976. **D.** Tertiary structure prediction of All0345. In both cases the HTH DNA binding domain is highlighted in green and the HTH DNA binding motif is highlighted with a black box in the secondary structure and in dark green in the tertiary structure. In the case of All0345 the AraC-E efector binding domain is highlighted in red.

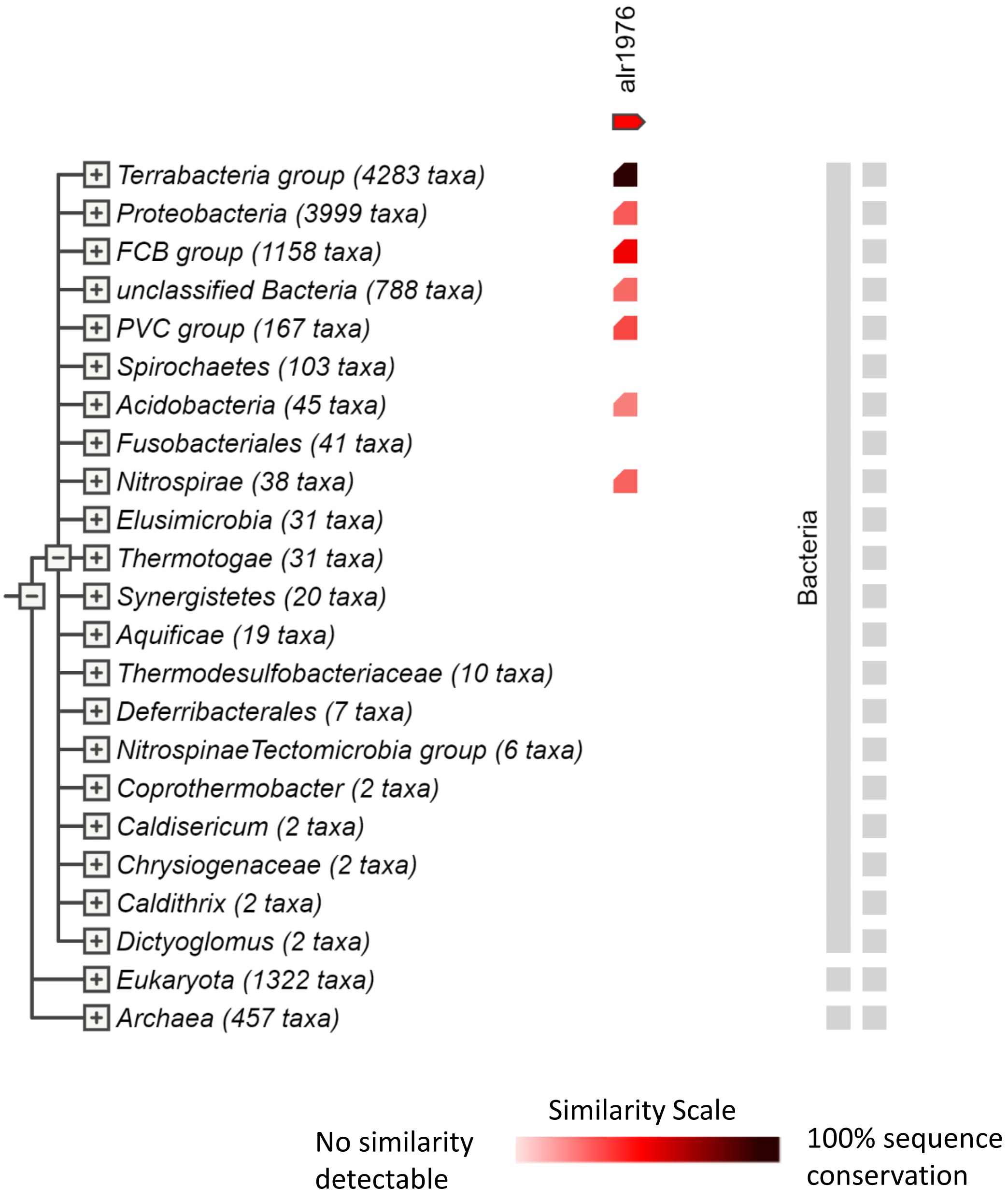

Figure S2. Occurrence of XRE-family regulator Alr1976/NsrX homologues in various bacterial species. Alr1976 is present across a broad range of bacterial phyla, as indicated by its presence in major groups including Terrabacteria, Proteobacteria, and FCB group. The STRING v11.5 database (string-db.org) was used to generate this representation

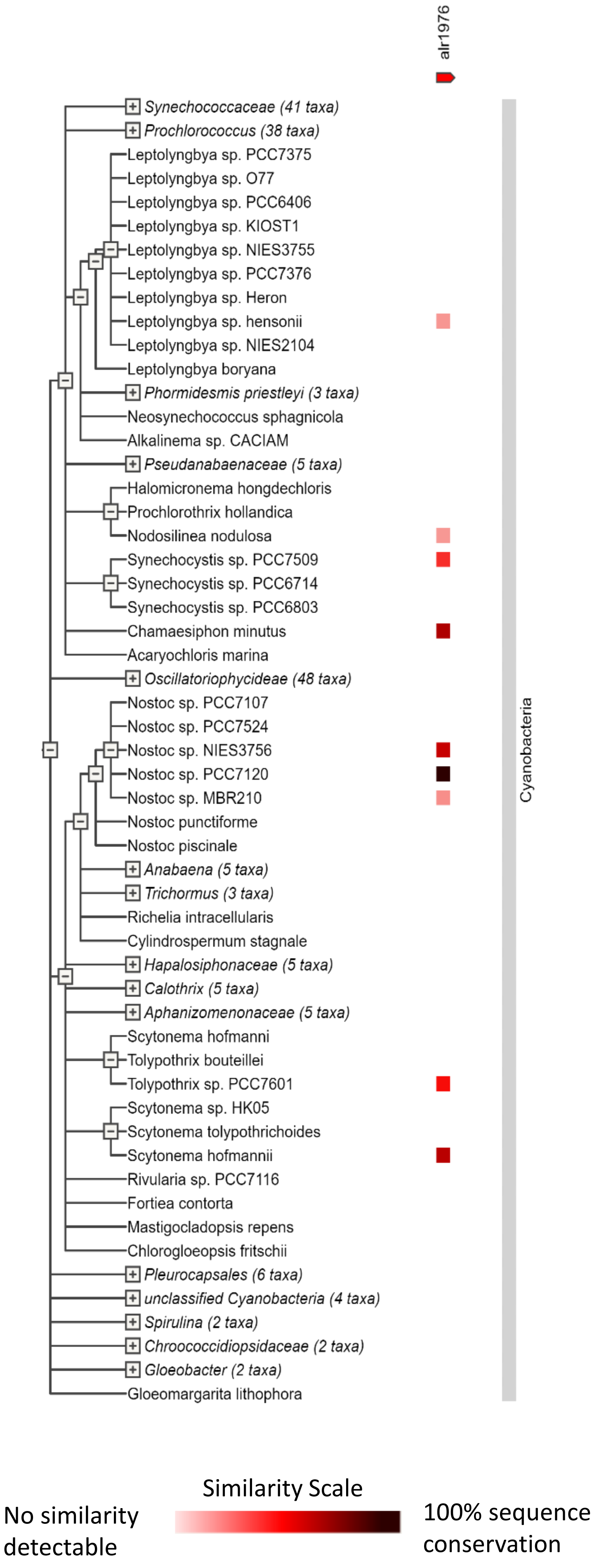

Figure S3. Occurrence XRE-family regulator Alr1976/NsrX homologues across cyanobacteria. The STRING v11.5 database (string-db.org) was used to generate this representation

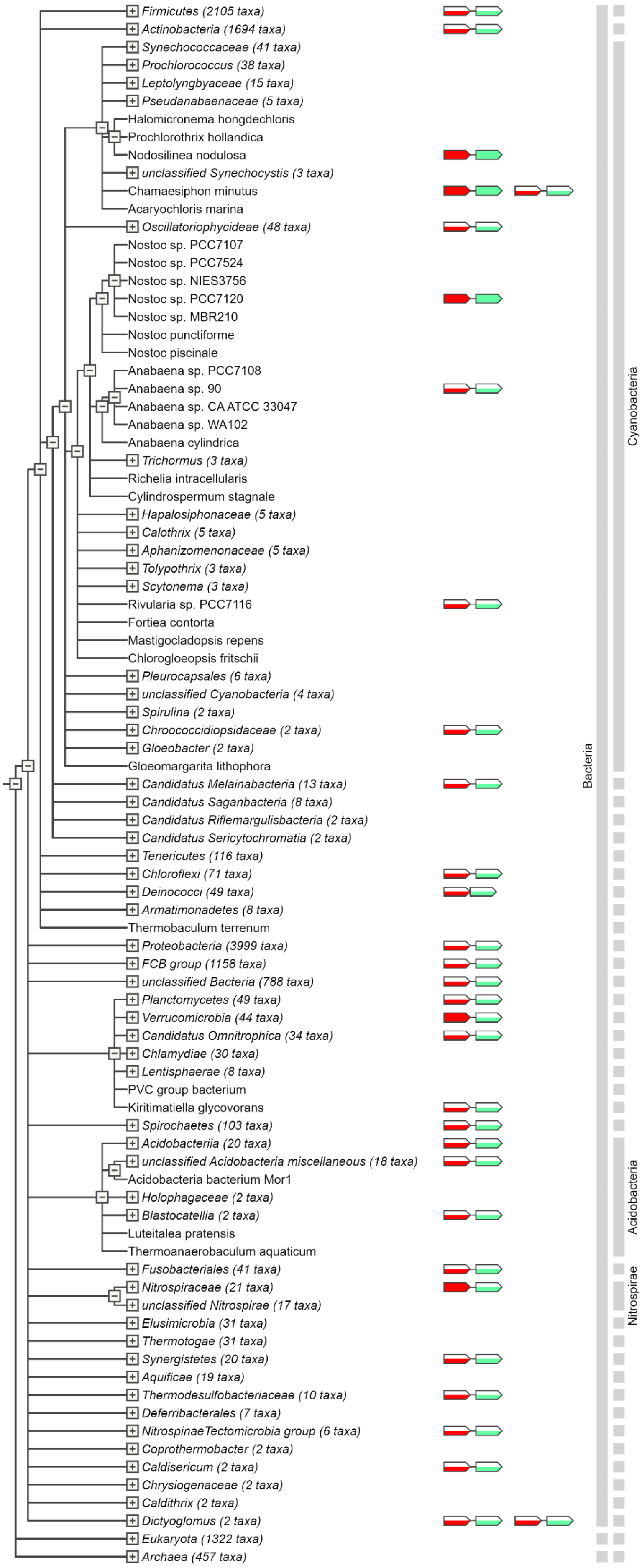

Figure S4. The arrangement of XRE-family regulator Alr1976/NsrX homologues (red arrows) with a Zn-metalloprotease homologue (green arrows) is conserved in various bacterial species. The STRING v11.5 database (string-db.org) was used to generate this representation

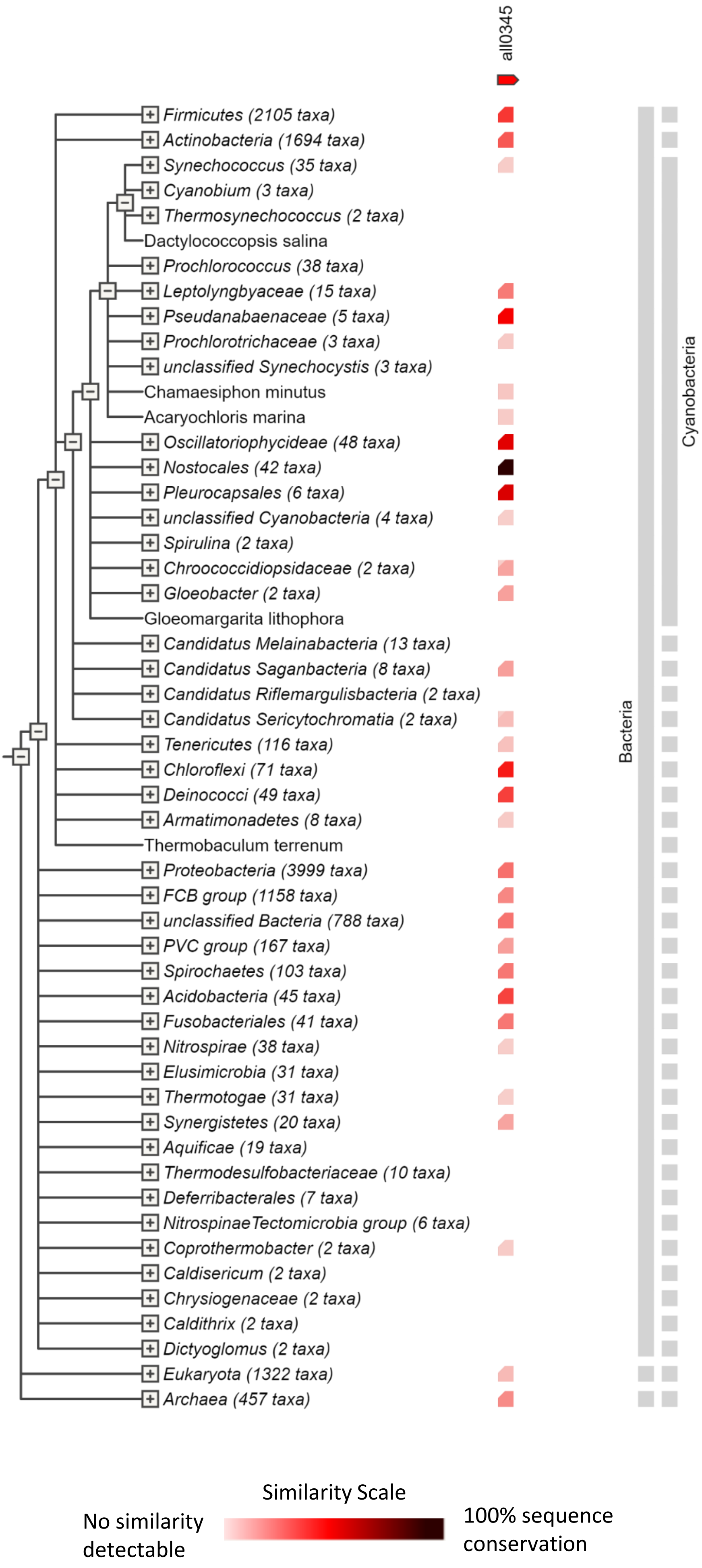

Figure S5. Global distribution of MerR-like All0345/NsrM homologues. The presence of All0345 homologues is observed across a broad range of bacteria, including a significant representation within Cyanobacteria. The STRING v11.5 database (string-db.org) was used to generate this representation

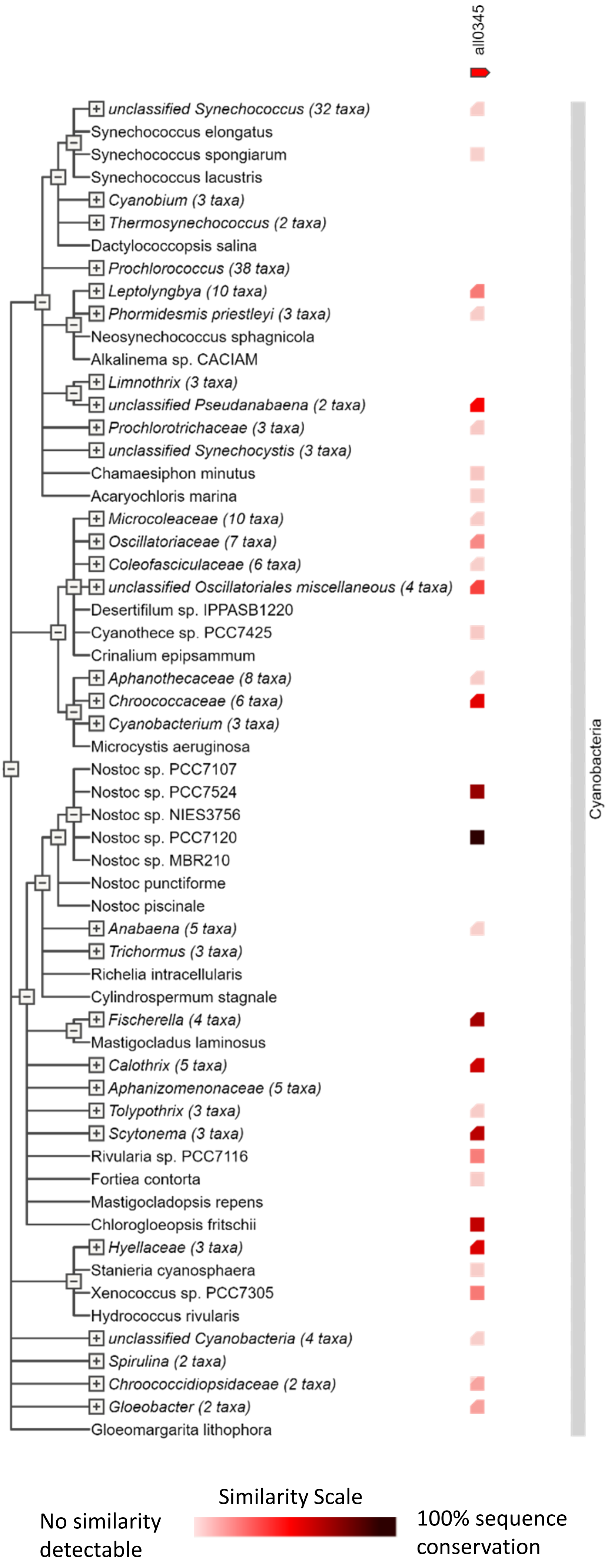

Figure S6. Distribution of All0345/NsrM homologues in cyanobacteria.
The STRING v11.5 database (string-db.org) was used to generate this representation.

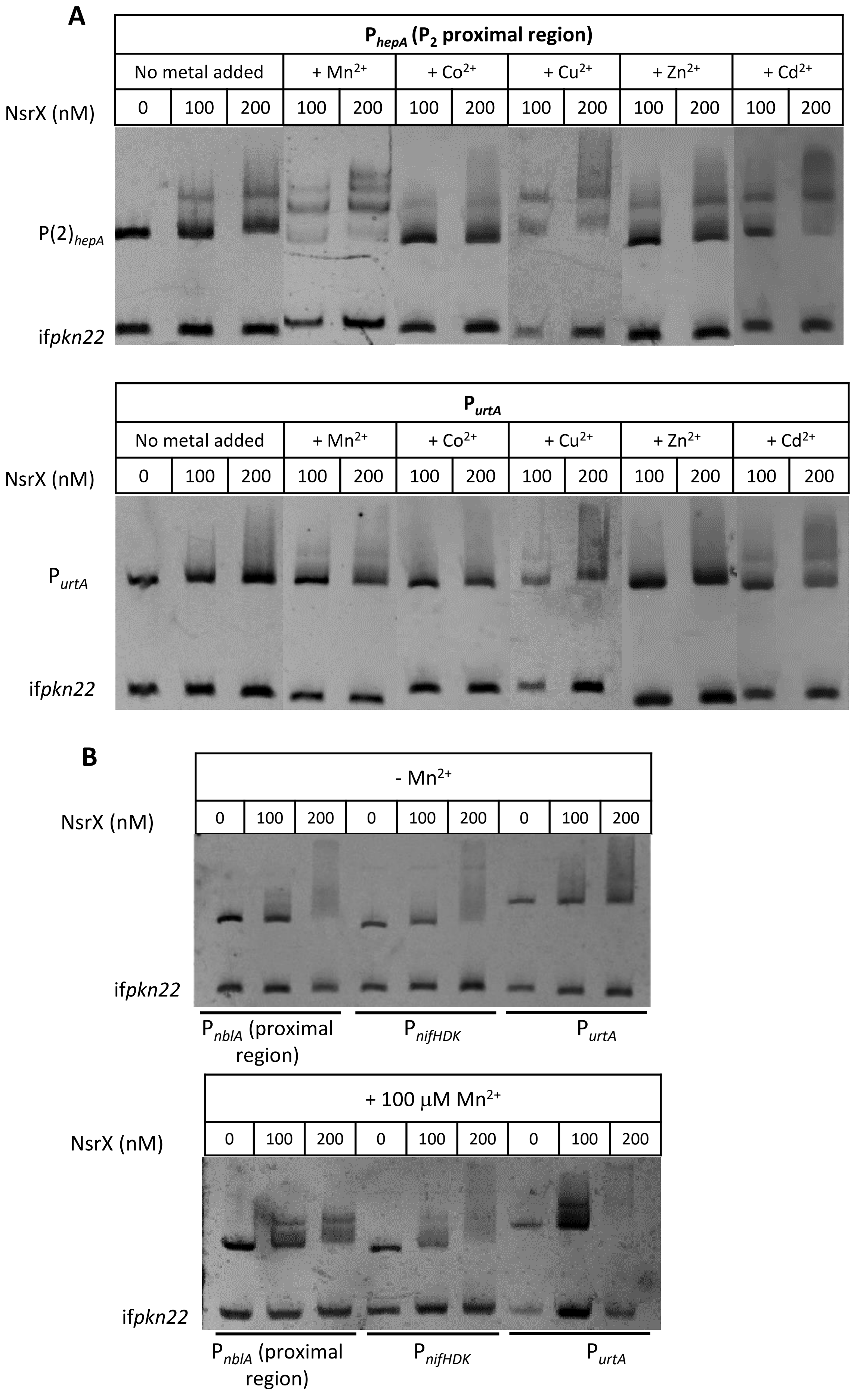

Figure S7. Modulation of the interaction of Alr1976 with DNA by metals. A. Electrophoretic Mobility Shift Assays (EMSA) showing the effect of different metals on the *in vitro* binding of Alr1976 to DNA. B. EMSA showing the interaction of Alr1976 *in vitro* with DNA in the presence or absence of manganese. In both cases, recombinant Alr1976 was incubated with different promoters in the presence or absence of 100 µM of metal ($MnCl_2$, $CoCl_2$, $CuSO_4$, $ZnSO_4$, $CdCl_2$). To analyze the effect of the different metals on DNA binding both gel and running buffer also included 100 µM of metal. Binding reactions were resolved by 6% PAGE. An internal fragment of the gene *pkn22* was used as non-specific competitor DNA.

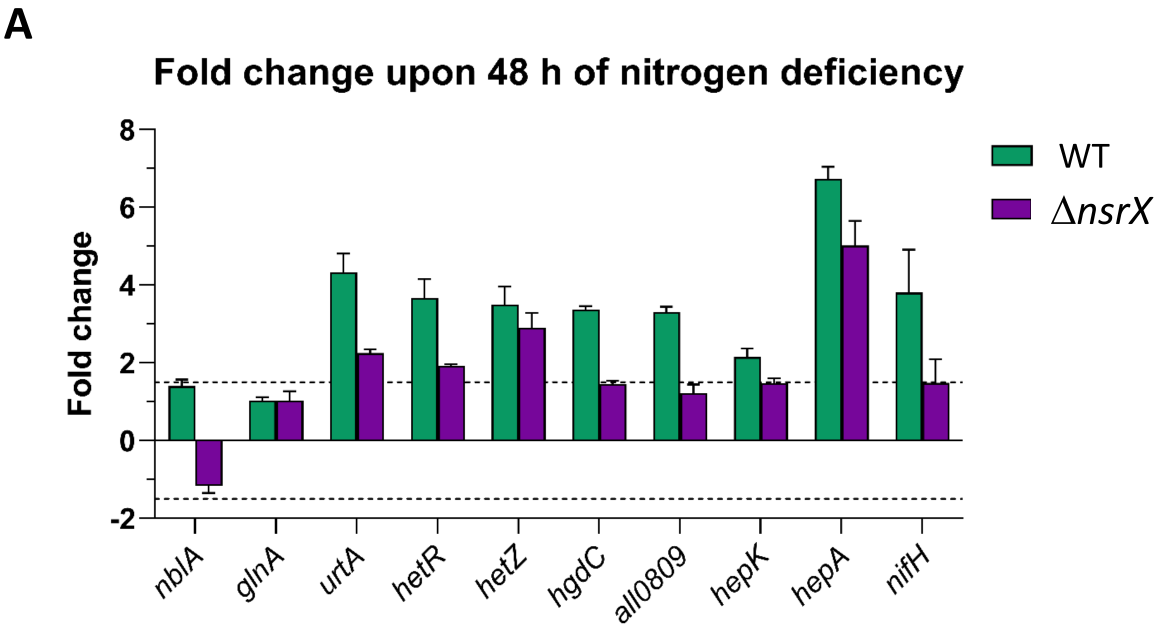

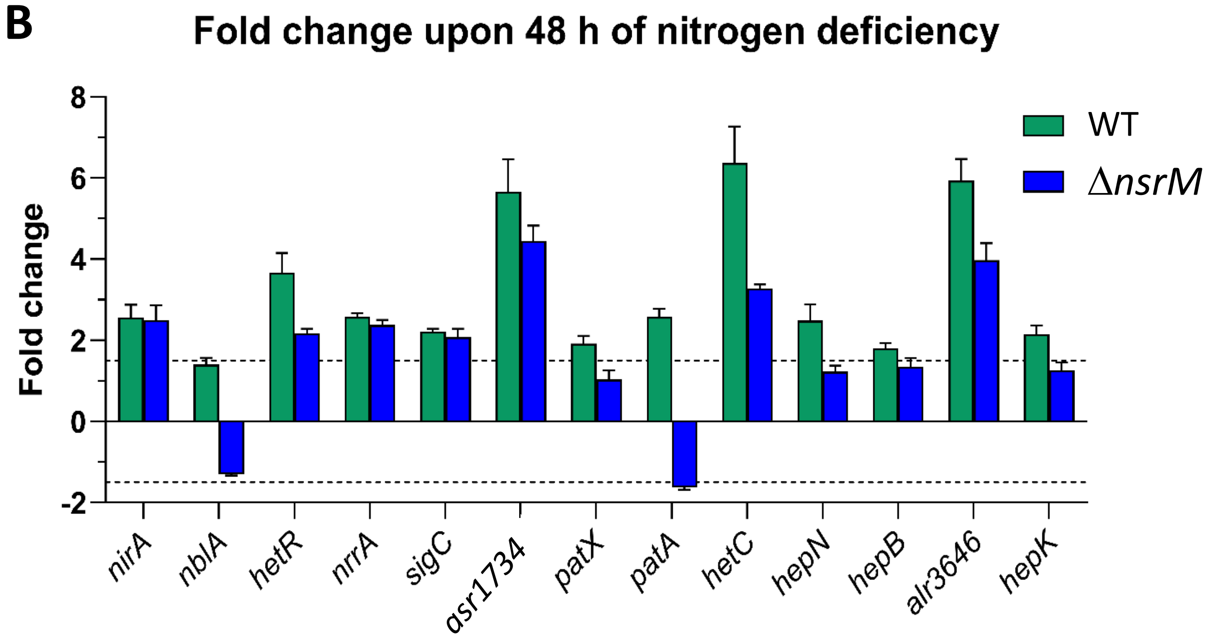

Figure S8. Differential expression of selected genes in *Anabaena* sp. PCC 7120 (WT) and the Δ*nsrX* (A) Δ*nsrM* (B) strains after 48 h of nitrogen step-down. Relative Real Time RT-PCR was used. Values are expressed as fold change (48 h after nitrogen step-down ($BG11_0$) vs. standard conditions (BG11)) and correspond to the average of three biological and three technical replicates. The standard deviation is indicated

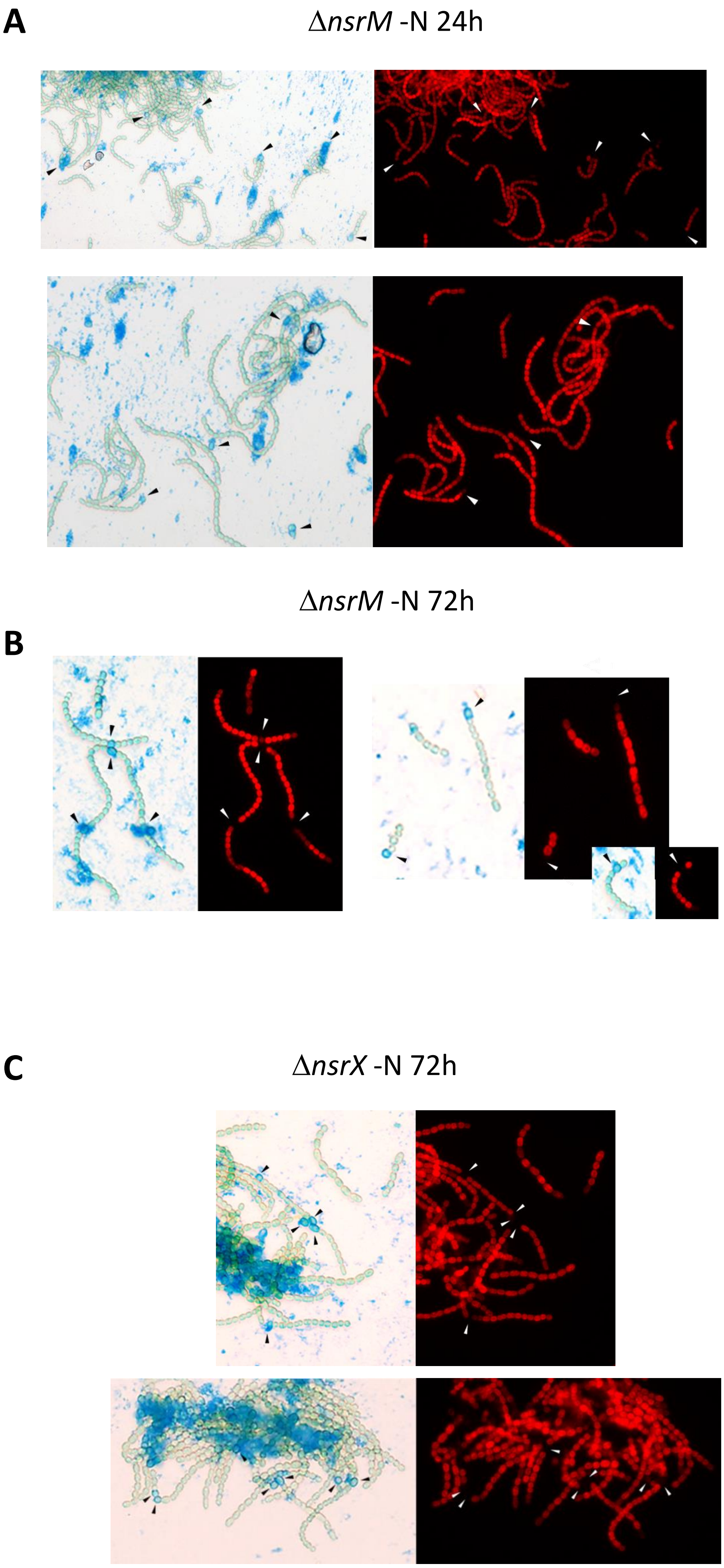

Figure S9. Representative bright field and fluorescence microscopy micrographs of Δ*nsrM* (A, B) and Δ*nsrX* (C) strains after 24 h (A) and 72 h (B, C) of nitrogen step-down and treated with Alcian Blue to stain the heterocyst polysaccharide layer. Heterocysts are marked with arrowheads.

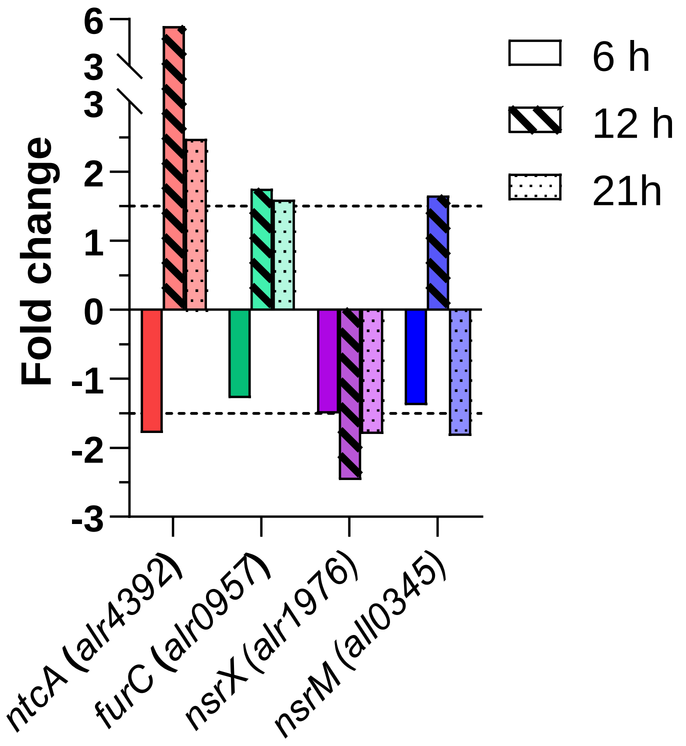

Figure S10. Transcriptional changes of *ntcA*, *furC*, *nsrX* and *nsrM* after 6, 12 and 21 hours of nitrogen step-down. Data ware obtained from Flaherty et al. 2011 (31)
