# Supplementary material for: NsrM (All0345) and NsrX (Alr1976), two FurC (PerR)-targeted transcriptional regulators, modulate nitrogen metabolism and heterocyst differentiation genes in the cyanobacterium Anabaena sp. strain PCC 7120
Source: Microbiol Spectr. 2025 Oct 13;13(11):e02311-25. doi: 10.1128/spectrum.02311-25 (PMC12584617; doi:10.1128/spectrum.02311-25)
Supplement: Files S1 and S2 — Supplemental methods for NsrX and NsrM purification and for construction of nsrM and nsrX deletion strains in Anabaena sp. PCC 7120. [file spectrum.02311-25-s0002.docx]

***Supplementary File S1. Supplementary methods for NsrX and NsrM purification***

Alr1976 and All0345 were obtained as His-tagged recombinant proteins. *nsrX* (*alr1976*) and *nsrM* (*all0345*) were amplified from *Anabaena* sp. PCC 7120 chromosomic DNA, using primers alr1976(NdeI)_pET28_up and alr1976(SalI)_pET28_dw for *alr1976* and primers all0345(NdeI)_pET28_up and all0345(SalI)_pET28_dw for *all0345* (Supplementary Table S1). The amplification products were digested with NdeI and SalI enzymes and cloned into the same restriction sites of pET-28a(+) plasmid (Novagen) followed by a His-tag, to obtain pET28-0345 and pET28-1976 plasmids, that were introduced into *E.* *coli* BL21 (DE3) cells. *E. coli* cells were grown in Luria–Bertani (LB) medium at 37°C until late exponential phase (OD_600_ 0.6–0.7). Recombinant protein production was induced with 1 mM isopropyl β-D-1-thiogalactopyranoside (IPTG) for 3 h at 37 ºC and cells were harvested by centrifugation.

NsrM and NsrX were purified by IMAC chromatography. Biomass of cells overexpressing His-tagged NsrM or NsrX were resuspended in purification buffer supplemented with 0,05 % Tween-20 and 1 cOmplete^TM^ ULTRA tablet of Protease Inhibitor Cocktail (Roche). Purification buffers consisted of 20 mM Bis-Tris pH 9, 10% glycerol in the case of NsrM and 50 mM Tris 50 pH 8, 300 mM NaCl, 10% glycerol in the case of NsrX. In both cases, cells were disrupted by sonication on ice for 4.5 min in periods of 45 s (amplitude 50 % (250 W), pulse ON 10 s, pulse OFF 30 s). Lysed cells were centrifuged at 4 °C at 125,000×g for 35 min and the resulting supernatant was filtered through 0.45 μM Millipore filters and loaded onto a Chelating Sepharose Fast Flow column previously loaded with 0.25 M NiSO_4_. Proteins were eluted using a linear gradient of 0–0.5 M Imidazol in purification buffer and fractions containing the elution peak were mixed. To remove metal traces, proteins were incubated with 1 mM EDTA for 20 min and subsequently dialyzed in the corresponding purification buffer. Purified proteins were analyzed by SDS/PAGE (Figure S11) and stored at −20°C.


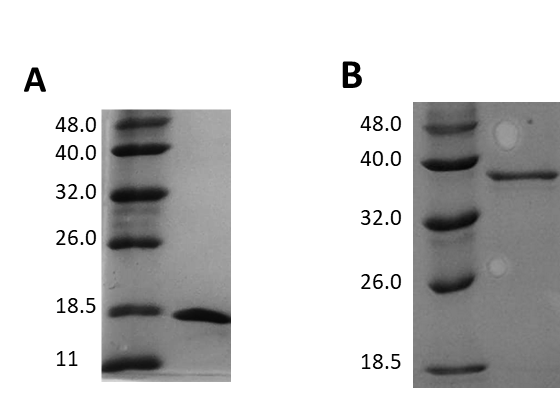


**Figure S11. Analysis of recombinant NsrX (A) and NsrM (B) after purification**. In each case, 2 μg of protein were loaded and resolved on 15% reducing SDS/PAGE gel and stained with Coomassie Blue.

***Supplementary File S2. Supplementary methods for* construction of *nsrM* and *nsrX* deletion strains in *Anabaena* sp. PCC 7120**

The *alr1976* and *all0345* deletion strains (∆*nsrX* and ∆*nsrM*) were constructed using CRISPR-Cpf1 genome editing based on the plasmid pSL2680 (Addgene 85581) using cloning methods and genome editing procedures reported previously (Ungerer & Pakrasi, 2016). Briefly, a guide RNA sequencing targeting *alr1976*/*nsrX* (5’- TAACTGGCTACAAGTACCAC-3’) or *all0345*/*nsrM* (5’- GAGTGGAAGCAAGACTCAAG-3’) were cloned into pSL2680 plasmid together with a homologous repair template composed of a region of ∼1 kb upstream from the coding sequence and a region of ∼1kb downstream from the coding sequence. To avoid side-off effects on the transcription of neighboring genes, in the case of *alr1976* a region of 200 bp of CDS was deleted, leaving 50 bp in the 5’ end of the CDS and 100 bp in the 3’ end of the CDS whereas in the case of *all0345* a region of 770 bp of CDS was deleted, leaving 100 bp in the 3’ end of the CDS (Figure S12). Cloning procedures were done in *E. coli* XL1-blue strain by using Gibson assembly after linearization of pSL2680 with BcuI and SalI restriction enzymes (ThermoFisher).


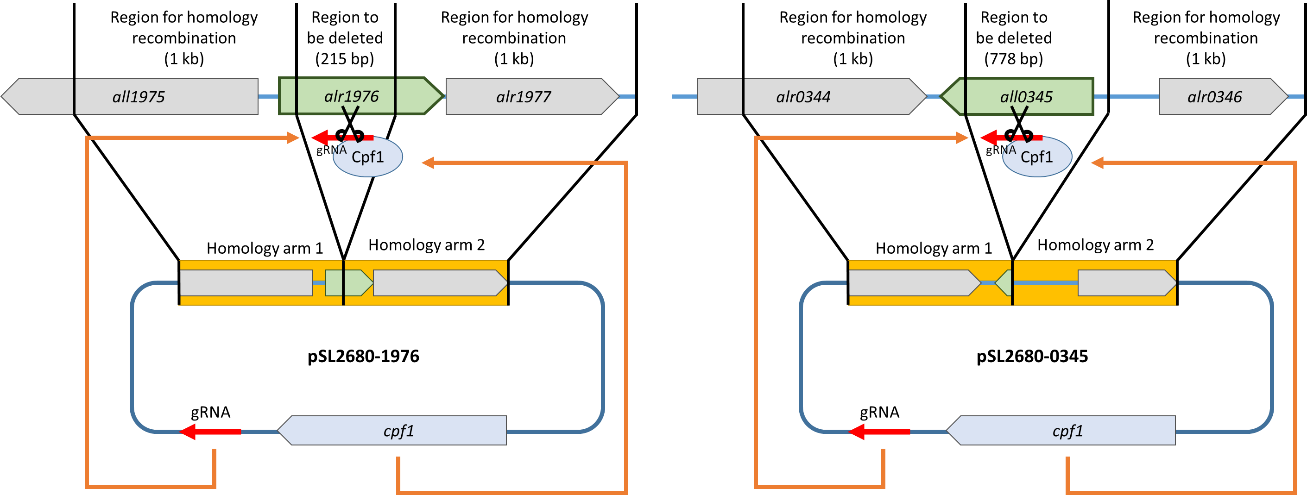


**B**

**A**

**Figure S12. Schematic representation of *nsrX*/*alr1976* (A) and *nsrM*/*all0345* (B) deletion using CRISPR-Cpf1.**

The resulting plasmid were named pSL2680-1976 and pSL2680-0345 and were introduced in *Anabaena* sp. PCC 7120 by triparental mating (Elhai *et al.*, 1997). Conjugation was performed with HB101 harbouring pRL443 and HB101 harbouring pRL623 + pSL2680-0345 or pSL2680-1976 as described by Ungerer & Pakrasi (2016). Briefly, 100 μL of overnight cultures of HB101 pRL443 and HB101 pRL623 +  pSL2680-0345/pSL2680-1976 were mixed with 200 μL of *Anabaena* sp. PCC 120 adjusted to an OD_750nm_ 0.8 and plated on BG11 agar supplemented with 5% Luria Broth. After 48 h incubation at 30 °C under 30  μE m^−2^ s^−1^ light, cells were collected using 1 mL of fresh BG11 and were transferred onto BG11 plates supplemented with 40 μg/mL neomycin. Colonies appeared within 8 days and were restreaked on BG11 plates supplemented with 40 μg/mL neomycin. *alr1976* (*nsrX*) or *all0345* (*nsrM*) deletion were checked using two pairs of PCR primers. One pair was used to check if the region to be deleted was absent in all copies of the chromosome and the other pair was used to check if the coding sequence of each gene was shortened as a consequence of genome editing (Figures S13 and S14). Fully segregated colonies were selected and the plasmid was cured by culturing cells in the absence of neomycin for 5 generations. Plasmid curation was checked by PCR using BcuI_F1 and leader_R1 primers.

Primers used for cloning and for checking *alr1976* (*nsrX*) or *all0345* (*nsrM*) deletion are included in Table S1.


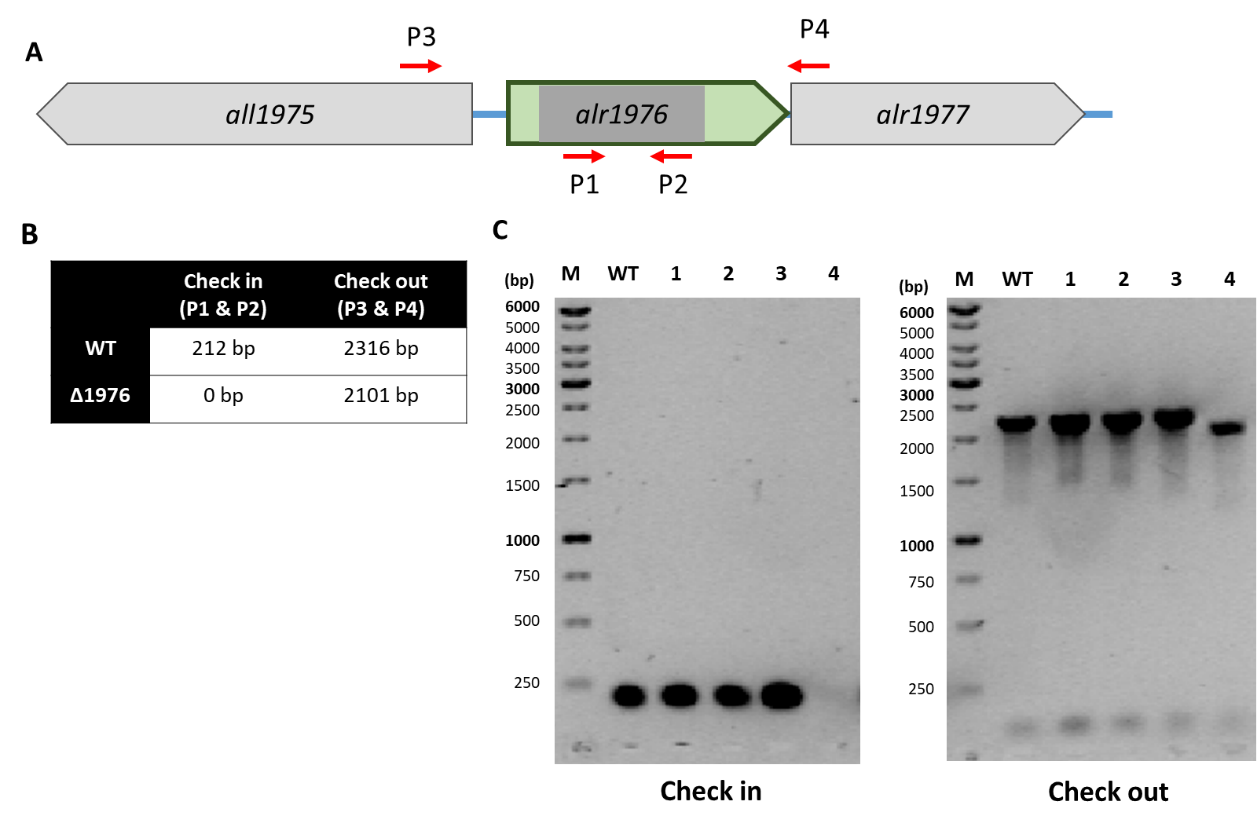


**Figure S13. Verification of *alr1976* (*nsrX*) deletion using CRISPR-Cpf1. A.** Relative positions of the oligonucleotides used for PCR deletion check. P1, P2, P3, P4 are short names for alr1976_in_check_F, alr1976_in_check_R, alr1976_out_check_F and alr1976_out_check_ R. The gray box represents the deleted region (lengths are not proportional). **B.** Expected sizes of the PCR products for WT and Δ*alr1976*. **C.** PCR verification on the genotype of four exconjugants randomly selected from the conjugation plates, including WT strain as control. Exconjugant 4 was selected as the Δ*alr1976* strain.


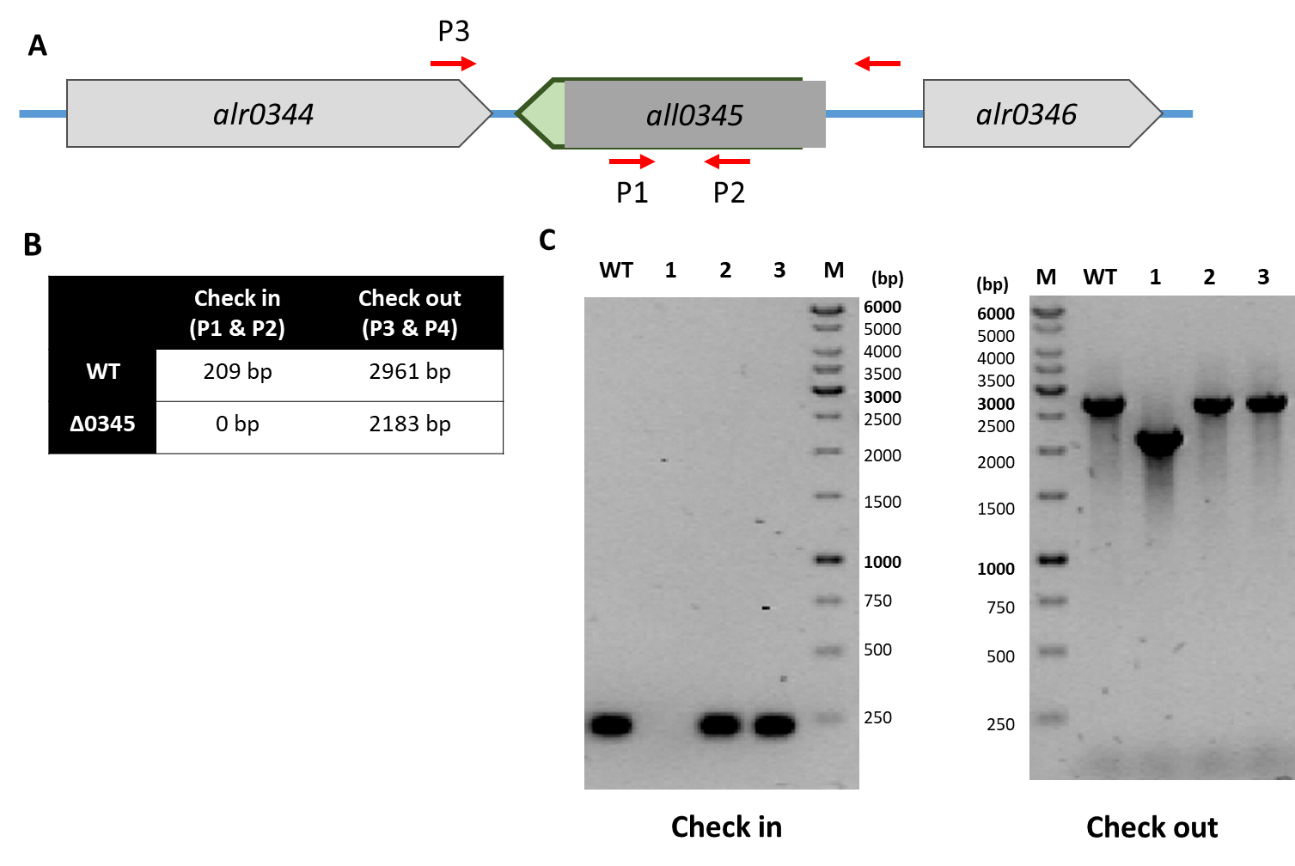


**Figure S14. Verification of *all0345* (*nsrM*) deletion using CRISPR-Cpf1. A.** Relative positions of the oligonucleotides used for PCR deletion check. P1, P2, P3, P4 are short names for all0345_in_check_F, all0345_in_check_R, all0345_out_check_F and all0345_out_check_ R. The gray box represents the deleted region (lengths are not proportional). **B.** Expected sizes of the PCR products for WT and Δ*all0345*. **C.** PCR verification on the genotype of four exconjugants randomly selected from the conjugation plates, including WT strain as control. Exconjugant 1 was selected as the Δ*all0345* strain.

**References**

1. Ungerer J, Pakrasi HB. 2016. Cpf1 Is A Versatile Tool for CRISPR Genome Editing Across Diverse Species of Cyanobacteria. Sci Rep 6:39681.

2. Elhai J, Vepritskiy A, Muro-Pastor AM, Flores E, Wolk CP. 1997. Reduction of conjugal transfer efficiency by three restriction activities of Anabaena sp. strain PCC7120. J Bacteriol 179:1998-2005.
