# Supplementary material for: NsrM (All0345) and NsrX (Alr1976), two FurC (PerR)-targeted transcriptional regulators, modulate nitrogen metabolism and heterocyst differentiation genes in the cyanobacterium Anabaena sp. strain PCC 7120
Source: Microbiol Spectr. 2025 Oct 13;13(11):e02311-25. doi: 10.1128/spectrum.02311-25 (PMC12584617; doi:10.1128/spectrum.02311-25)
Supplement: Table S1 — Oligonucleotides used in this study. [file spectrum.02311-25-s0003.docx]

## Supporting information

The following Supporting information is available for this article:

**Table S1.** Oligonucleotides used in this study.

| **Table S1.** Oligonucleotides used in this study | | |
| --- | --- | --- |
| **EMSA** |  |  |
| **Primer** | **Sequence (5´→ 3´)** | **Purpose** |
| Pif*pkn*_up | AAAGATGAATTACACTGGCG | Internal fragment of *pkn22* (EMSA negative control) |
| Pif*pkn*_dw | CTGCAAACTGTGGCAGAATA |  |
| Palr1976_fw | ACGCGGATATGGTACAGCAG | *alr1976 (nsrX)* promoter |
| Palr1976_rv | ACTAGGCTCGCCAAGCGTTC |  |
| Pall0345_fw | AAATGACACGGTAGTCGCTG | *all0345 (nsrM)* promoter |
| Pall0345_rv | ACTGCGCTGTAGGTGATATG |  |
| P1nir_fw | TTCACTATTAGAGCATCGATTC | *alr0607-12 (nirA-nrtABCD-narB)* promoter (proximal region) |
| P1nir_rv | GCTTTGAATTTCTCAAACTTATTG |  |
| P2nir_fw | AATCCCGACGTTTCATAGCC | *alr0607-12 (nirA-nrtABCD-narB)* promoter (distal region) |
| P2nir_rv | TTTGGCTCCCACTTCCCTAC |  |
| PurtA_fw | GGAGCTTGATTGGATATGTG | *all1951-47 (urtABCDE)* promoter |
| PurtA_rv | TCTGCGTCGGTTAATTCGTC |  |
| PglnA_fw | TGTTACTGCATCGCGCATTCC | *alr2328 (glnA)* promoter |
| PglnA_rv | CGAAACAAAGTTGATGAC |  |
| P1nblA_fw | GAACAGTTTTGAATAGGTAG | *asr4517 (nblA)* promoter (proximal region) |
| P1nblA_rv | CATAACAGACTCCTAAAAGAC |  |
| P2nblA_fw | CGCTTTAGAATAACGTTCCC | *asr4517 (nblA)* promoter (distal region) |
| P2nblA_rv | TTTTGCACCTGAGCCTACTG |  |
| PntcA_fw | CATGGTTAGCAAAAATGATG | *alr4392 (ntcA)* promoter |
| PntcA_rv | CTTGTGTCACGATCATCTCC |  |
| P1hetR_fw | GGGAAAGTCCTTGTAGGTTAC | *alr2339 (hetR)* promoter  (proximal region) |
| P1hetR_rv | CGCTTGATCAGATCGATG |  |
| P2hetR_fw | GTCTATAATTTTCCCTCCAG | *alr2339 (hetR)* promoter  (middle region) |
| P2hetR_rv | CTAATAAGTAACCTACAAG |  |
| P3hetR_fw | AAGGTTAATATGACAAAGGAC | *alr2339 (hetR)* promoter  (distal region) |
| P3hetR_rv | CTGGAGGGAAAATTATAG |  |
| PnrrA_fw | GCCAAAAATTAACTCTGTG | *all4312 (nrrA)* promoter |
| PnrrA_rv | CAACGATTTCAATACAAACC |  |
| PhanA_fw | GGTGATGACAAGGCTTGG | *asr3935 (hanA)* promoter |
| PhanA_rv | CATTTGGAACTCCTTAATGTTTAC |  |
| P1asr1734_fw | GTTGATGTGAAAAGCTTTAAAAAC | *asr1734* promoter (proximal region) |
| P1asr1734_rv | CATAGTTTTAGAGATTCCTGTGC |  |
| P2asr1734_fw | GATTTTACTTTTGATAAAATTGCTC | *asr1734* promoter  (distal region) |
| P2asr1734_rv | CGAAGATTGAATCTTCCTGTGTC |  |
| Pall1692_fw | CCCATGTAGATGTGACTATGC | *all1692 (sigC)* promoter |
| Pall1692_rv | GTTGCTGGCATAAGATCGTC |  |
| P1alr4249_fw | GATTGCAGTTATGTCTCTCAACG | *alr4249 (sigE)* promoter  (proximal region) |
| P1alr4249_rv | CCTTGAGGGATTCATGCTTTG |  |
| P2alr4249_fw | GCCTTAAACTCAGTCCGTAAAGTTC | *alr4249 (sigE)* promoter  (distal region) |
| P2alr4249_rv | CGTTGAGAGACATAACTGCAATC |  |
| P1alr3280_fw | TTCTATGAAAACCAGTTGCAGAG | *alr3280 (sigG)* promoter  (proximal region) |
| P1alr3280_rv | AGAGTTTGTCAACTTGCACCGAT |  |
| P2alr3280_fw | CCTGGTATCCGACTTAAAAG | *alr3280 (sigG)* promoter  (distal region) |
| P2alr3280_rv | CAATACGCAGATTGTTATTTAC |  |
| P*patA*_fw | GGTTTGAGCCAATAACTATACGTG | *all0521 (patA)* promoter |
| P*patA*_rv | GTAATCGGAAGTGTTTTCATGG |  |
| P1patB_fw | TCAAATGTTCGCAAGCGAAG | *all2512 (patB)* promoter (proximal region) |
| P1patB_rv | TGTCACATCCAACGCAACTG |  |
| P2patB_fw | AGTTGACTTCTGTAGCCAAG | *all2512 (patB)* promoter (distal region) |
| P2patB_rv | GCACTAGAGCCTAAGATTAC |  |
| P1patS_fw | GCATATTTACGTAAAAAGTTCTATC | *asl2301 (patS)* promoter (distal region) |
| P1patS_rv | CCGGATAGTTAAATGCAAAAAG |  |
| P2patS_fw | GCATTTAACTATCCGGATTTTTTAC | *asl2301 (patS)* promoter (middle region) |
| P2patS_rv | GCACCATTGTTATTCTTATTGACC |  |
| P3patS_fw | GGTCAATAAGAATAACAATGGTGC | *asl2301 (patS)* promoter (proximal region) |
| P3patS_rv | CATAATCTTAAAATCGGTGAATTAC |  |
| PpatX_fw | AAGCAACATCTGACGGTTCG | *asl2332 (patX)* promoter |
| PpatX_rv | TCTGGTTGATTACGTTTGGG |  |
| P1hetC_fw | CAGATGTCGATGTTGCTCAC | *alr2817 (hetC)* promoter (proximal region) |
| P1hetC_rv | CATAGTTTAATTTCTGTTTGGTG |  |
| P2hetC_fw | GCTACTAGAAATGAGGAGAGGG | *alr2817 (hetC)* promoter (distal region) |
| P2hetC_rv | GTGAGCAACATCGACATCTG |  |
| P1hetN_fw | GTTATGAGTGAGTTATCCTGG | *alr5358 (hetN)* promoter (proximal region) |
| P1hetN_rv | CATTGTAACCTGCTAGTCTCC |  |
| P2hetN_fw | GGAGAAGACGCGATGAATCG | *alr5358 (hetN)* promoter (distal region) |
| P2hetN_rv | CTAGCTCATAGAACTTACGCGC |  |
| PhetL_fw | ATGCTGGTTATACCCAAGAC | *all3740 (hetL)* promoter |
| PhetL_rv | AGTTGCTCTTACCAAGTCGC |  |
| P1hetP_fw | GGGCAGTTAATAGATGAAGC | *alr2818 (hetP)* promoter (proximal region) |
| P1hetP_rv | AGTTGGTTATGCCTGTAGTG |  |
| P2hetP_fw | TCTCAAATGCACAGTAGGCG | *alr2818 (hetP)* promoter (distal region) |
| P2hetP_rv | GCTTCATCTATTAACTGCCC |  |
| PhetZ_fw | GCGTTTAGTTTATCCGCAAA | *alr0099 (hetZ)* promoter |
| PhetZ_rv | CTCAAGCATTGTTGTAGCCG |  |
| P1hepA_fw | CAGTATCTGTAGGGTGCCTATC | *alr2835 (hepA)* promoter (distal region) |
| P1hepA_rv | CTTGCTTGCTGAAATAAGTATAG |  |
| P2hepA_fw | GGGTGAATAATGCCCTG | *alr2835 (hepA)* promoter (proximal region) |
| P2hepA_rv | CTCTTTCCAGAAGCTATTAGC |  |

| P1hepB_fw | CCCTAGAGGATGAAAATTGAAG | alr3698 (hepB) promoter (distal region) |
| --- | --- | --- |
| P1hepB_rv | GGCATTTCTCTCATGATATAGAG |  |
| P2hepB_fw | CTCTATATCATGAGAGAAATGCC | a*lr3698 (hepB)* promoter (proximal region) |
| P2hepB_rv | CTCCTAGTACTGTATGGAGCTTG |  |
| P1hepC_fw | GCCAATTAAAGTTGTCATGATTAAG | *alr2834 (hepC)* promoter distal region |
| P1hepC_rv | CCTAACCTTTGTAATATCCGCC |  |
| P2hepC_fw | GGCGGATATTACAAAGGTTAG | *alr2834 (hepC)* promoter proximal region |
| P2hepC_rv | CGCTTGTCATAATGTCTTTGG |  |
| PhepK_fw | CGCTGGTTATGAAGCTGG | *all4496 (hepK)* promoter |
| PhepK_rv | CCCTCAAATCTCCTTACCCTC |  |
| PhglT_fw | TTATGGCTGGGCGATGATAG | *all5341 (hglT)* promoter |
| PhglT_rv | TAAACAGCGCAATGCGAAGC |  |
| Pall5346_fw | CGCAGATAGCGAACGGGTAG | *alr5346 (hgdC)* promoter |
| Pall5346_rv | CATCTTGGAAACCAAGTTGC |  |
| PhgdD_fw | CGTTTTGAAGACAAACAGGG | *alr2887 (hgdD)* promoter |
| PhgdD_rv | TGGACTGTAGCATTAGGCAG |  |
| PdevB_fw | CGTTATCAATAGAGTCCATAGCC | *all0809-07* promoter |
| PdevB_rv | CACAATCCCATACCTTTGTAG |  |
| Palr3646-49_fw | CTTTCTATACGGTGTCAAGTTTAC | *alr3646-49* promoter |
| Palr3646-49_rv | GTTCTTTCCGATATTGGTCATG |  |
| Palr4973-75_fw | CACCTCGGACACAATCAAAC | *alr4973-75* promoter |
| Palr4973-75_rv | GAGATTCCTGAATCGGTCTG |  |
| PhenR_fw | TTCCTTGAACCAAGATTTGC | *alr1085-86 (alr1085-henR)* promoter |
| PhenR_rv | AACAAGCACCCAGTTACCTC |  |
| PdevR_fw | GTGGAGAGAAGGTTTAACTG | *alr0442 (devR)* promoter |
| PdevR_rv | ATGTTTCACGCCTAAGCCAC |  |
| PdevH_fw | GGTTTTTATGAGGGGCTAAG | *alr3952 (devH)* promoter |
| PdevH_rv | GATGGAGATTGCATAGGTAAC |  |
| PhepS_fw | ATTGATTGGCTTGCACGGAG | *all2760 (hepS)* promoter |
| PhepS_rv | CGTCCGAGTTCTTGAATAAC |  |
| PhepN_fw | TTGGCAAATCTTGCGGCAAG | *alr0117 (hepN)* promoter |
| PhepN_rv | AGCAGTGGCATTTGTAGACC |  |
| P*nifH_fw* | GGCGTGTGGCTCTTGTTC | *all1455-40 (nifHDK)* promoter |
| P*nifH_rv* | GGTTGGTTAGTTGGTCGG |  |
| PxisHI_fw | GCAGAATGTGGCTTTACTAC | *alr1461-62 (xisHI)* promoter |
| PxisHI_rv | GAGCTGTTCTCACGATGTTATG |  |
| Palr1174_fw | CATGGATGCCAGCGATAGATG | *alr1174 (rbrA)* promoter |
| Palr1174_rv | GCTTCTAAGTTTTGCAGTGTAG |  |

| **RT-PCR** | |  | |  |
| --- | --- | --- | --- | --- |
| **Primer** | | **Sequence (5´→ 3´)** | | **Purpose** |
| rnpB_for | | AGCGGAACTGGTAAAAGACCA | | *rnpB* housekeeping |
| rnpB_rev | | GAGAGGTACTGGCTCGGTAAAC | |  |
| RT_alr1976_up | | GCCAATGCTTTAGCGTCCTTA | | *alr1976 (nsrX)* |
| RT_alr1976_dw | | CGTCATCTTGTTGCGAAAGGT | |  |
| RT_all0345_up | | TCGCTACTACTCGGCTGAACAA | | *all0345 (nsrM)* |
| RT_all0345_dw | | CTAGGGAAAACCCCAAATCCTT | |  |
| RT_hetR fw | | CGCTATGCGAGCCTTAGAAGA | | *alr2339 (hetR)* |
| RT_hetR rv | | CAGTTCCTGCATGGCTTCATC | |  |
| RT_hetZ fw | | CTGCGGGCGCAAACTT | | *alr0099 (hetZ)* |
| RT_hetZ rv | | CGGCGGCTTGTTCGATAT | |  |
| RT_all1692_fw | | TGATTTGTCGGTGGATGAGATC | | *all1692 (sigC)* |
| RT_all1692_rv | | ACGACGGTTAGCGCTAGCA | |  |
| RT_hepA fw | | CGCGGTGTCCGTTTATCTG | | *alr2835 (hepA)* |
| RT_hepA rv | | TGGTGGCTTCGTCAAGAATG | |  |
| RT_hepB_up | | GCCCAGTCAATCTTTTGAAGGT | | *alr3698 (hepB)* |
| RT_hepB_dw | | AACTGTGGTGAAAATGGTGTTAAAAT | |  |
| RT_hepK fw | | CGAACCCAAACCCAGTTGAC | | *all4496 (hepK)* |
| RT_hepK rv | | GTTTAGCAATTAGCCGCCACTT | |  |
| 5346qPCR_fw | | TCCACGTTTCTCCGCATCA | | *all5346 (hgdC)* |
| 5346qPCR_fw | | GATCAGCACCTGGTTTAAGAGTGA | |  |
| RT_hepN_up | | TGGCGGTGGATCGTAATTTAA | | *alr0117 (hepN)* |
| RT_hepN_dw | | TGCATCTGCCAAAACATAAATCTC | |  |
| RT_hetC fw | | GCTTACAGCACCAGCCATAGC | | *alr2817 (hetC)* |
| RT_hetC rv | | GACCGTGATTTGTGTTGTTTGAC | |  |
| RT_asr1734_up | | AATTCCGGTTAGTTGAGGAAAAAA | | *asr1734* |
| RT_asr1734_dw | | GATCCTTCCGTTAGGGCATCT | |  |
| RT_alr3646_up | | CATGACCAATATCGGAAAGAACTG | | *alr3646-49 (devBCA)* |
| RT_alr3646_dw | | GCGTACCTGTAGAAACATTCAAACC | |  |
| RT_patA_up | | CAACAGCATGGGCGTGTTC | | *all0521 (patA)* |
| RT_patA_dw | | TGTTAACTGGTATCTGGTTATGTTCTGTAG | |  |
| RT_patX_up | | CGATTTCGCTTTTTCTCCCTCTA | | *asl2332 (patX)* |
| RT_patX_dw | | CGTCCAGTACCGCGATGTG | |  |
| RT_devB_up | | GACTTGCAGCGCATCCAA | | *all0809-07(devBCA)* |
| RT_devB_dw | | TGATTTGTGCTAATGTGGCTTTG | |  |
| RT_nifH_up | | TGCGCCGTATGACCGTTA | | *all1455-54-40 (nifHDK)* |
| RT_nifH_dw | | GCTAATGCGCGGTACTCTTGA | |  |
| RT_nblA_up | | TTCAGCATTCGCTCATTTGC | | *asr4517 (nblA)* |
| RT_nblA_dw | | CTTACGGACAACCATTTGTTC | |  |
| RT_urtA_up | | GCGCTAATAGCACCCCAAAC | | *all1951-47 (urtABCDE)* |
| RT_urtA_dw | | CCACTAGCAGCCACAGGAGAA | |  |
| RT_glnA_up | | GCACTGTCACCAATCTATCTGGAA | | *alr2328 (glnA)* |
| RT_glnA_dw | | CCCATTTCACTTAAGCCAGCAT | |  |
| RT_nir-nrt-nar fw | | GACAGATATCGAAGTCATCAAGCAA | | *alr0607-12  (nirA-nrtABCD-narB)* |
| RT_nir-nrt-nar rv | | CACCAGATGAGCCAATTTTTAAGTT | |  |
| **pSL2680 cloning** | | | | |
| **Primer** | | **Sequence (5´→ 3´)** | | |
| BcuI_F1 | | GCAGCTATTTGCGGTGAGAGC | | |
| leader_R1 | | CCGTTTTTGCCTAAATCAGCGC | | |
| leader_F2 | | CGCTGATTTAGGCAAAAACGGGTCTAAGAACTTTAAATAATTTCTAC | | |
| gRNA_all0345_R2 | | CTTGAGTCTTGCTTCCACTCAtctacaacagtagaaattatttaaag | | |
| gRNA_all0345_F3 | | GAGTGGAAGCAAGACTCAAGgtctaagaactttaaataatttctac | | |
| all0345_Ter_R3 | | AGTCGTGCAGATGCGCGGTACCaactaccgcattaaag | | |
| all0345_Up_F4 | | cggtagttGGTACCGCGCATCTGCACGACTATATTC | | |
| all0345_Up_R4 | | TAGGAGCAGTAATATCCCTAAGGGTAGAGTCAAGAG | | |
| all0345_Dw_F5 | | ACTCTACCCTTAGGGATATTACTGCTCCTAACCGCG | | |
| all0345_Dw_R5 | | tgcgctgcccggattacagatcctctagagTCGGGAAGATAGCAAAACGGG | | |
| gRNA_alr1976_R2 | | GTGGTACTTGTAGCCAGTTAAtctacaacagtagaaattatttaaag | | |
| gRNA_alr1976_F3 | | TAACTGGCTACAAGTACCACgtctaagaactttaaataatttctac | | |
| alr1976_Ter_R3 | | TTCATCTTTACCCTGCGGTACCaactaccgcattaaag | | |
| alr1976_Up_F4 | | cggtagttGGTACCGCAGGGTAAAGATGAATGGCGC | | |
| alr1976_Up_R4 | | GCTGGATCAAGTTTTCTTTTTGCGAACTAGGCTCGC | | |
| alr1976_Dw_F5 | | CTAGTTCGCAAAAAGAAAACTTGATCCAGCGATCGC | | |
| alr1976_Dw_R5 | | tgcgctgcccggattacagatcctctagagTTACGCCAACGGTTTCGTTTG | | |
| **Deletion check** | | | | |
| **Primer** | | **Sequence (5´→ 3´)** | | |
| all0345_out_check_F | | ACAATAACCCCAAATGGGCAG | | |
| all0345_out_check_R | | GTGTAGCGCAAGCTCAAACTG | | |
| all0345_in_check_F | | TTGTGATCAAAAAGGTTGCCC | | |
| all0345_in_check_R | | GCCTTCAATTGAGATGACAGC | | |
| alr1976_out_check_F | | TTGGTCACAGAAGCACAGTTG | | |
| alr1976_out_check_R | | AGGAACCAAACGGGAAAACAG | | |
| alr1976_in_check_F | | GAGGTAGTAGAGGGTTGCGAG | | |
| alr1976_in_check_R | | GTCGGCTCGTAATTGAATTGC | | |
| **pET28 cloning** | | | | |
| **Primer** | | **Sequence (5´→ 3´)** | | |
| all0345_pET28_up(NdeI) | | GGAATTCCATATGtactgtagacagttggatagattaag | | |
| all0345_pET28_dw(SalI) | | TTTGTCGACtcaagcttgagctactggaaactg | | |
| alr1976_pET28_up(NdeI) | | GGAATTCCATATGggaagttatctagatatagaacgc | | |
| alr1976_pET28_dw(SalI) | | TTTGTCGACttatgggtcttgcttcaattcgtc | | |
